# Image resampling and discretization effect on the estimate of myocardial radiomic features from T1 and T2 mapping in hypertrophic cardiomyopathy

**DOI:** 10.1038/s41598-022-13937-0

**Published:** 2022-06-17

**Authors:** Daniela Marfisi, Carlo Tessa, Chiara Marzi, Jacopo Del Meglio, Stefania Linsalata, Rita Borgheresi, Alessio Lilli, Riccardo Lazzarini, Luca Salvatori, Claudio Vignali, Andrea Barucci, Mario Mascalchi, Giancarlo Casolo, Stefano Diciotti, Antonio Claudio Traino, Marco Giannelli

**Affiliations:** 1 Unit of Medical Physics, Pisa University Hospital “Azienda Ospedaliero-Universitaria Pisana”, Via Roma 67, 56126 Pisa, Italy; 2 Unit of Radiology, Azienda USL Toscana Nord Ovest, Apuane Hospital, 54100 Massa, Italy; 3 Institute of Applied Physics “Nello Carrara”, Italian National Research Council, 50019 Sesto Fiorentino, Italy; 4 Unit of Cardiology, Azienda USL Toscana Nord Ovest, Versilia Hospital, 55041 Lido di Camaiore, Italy; 5 Unit of Radiology, Azienda USL Toscana Nord Ovest, Versilia Hospital, 55041 Lido di Camaiore, Italy; 6 Department of Experimental and Clinical Biomedical Sciences “Mario Serio”, University of Florence, 50121 Florence, Italy; 7 Department of Electrical, Electronic, and Information Engineering “Guglielmo Marconi”, University of Bologna, 47522 Cesena, Italy

**Keywords:** Data processing, Image processing, Diagnostic markers, Prognostic markers, Magnetic resonance imaging, Cardiomyopathies

## Abstract

Radiomics is emerging as a promising and useful tool in cardiac magnetic resonance (CMR) imaging applications. Accordingly, the purpose of this study was to investigate, for the first time, the effect of image resampling/discretization and filtering on radiomic features estimation from quantitative CMR T1 and T2 mapping. Specifically, T1 and T2 maps of 26 patients with hypertrophic cardiomyopathy (HCM) were used to estimate 98 radiomic features for 7 different resampling voxel sizes (at fixed bin width), 9 different bin widths (at fixed resampling voxel size), and 7 different spatial filters (at fixed resampling voxel size/bin width). While we found a remarkable dependence of myocardial radiomic features from T1 and T2 mapping on image filters, many radiomic features showed a limited sensitivity to resampling voxel size/bin width, in terms of intraclass correlation coefficient (> 0.75) and coefficient of variation (< 30%). The estimate of most textural radiomic features showed a linear significant (p < 0.05) correlation with resampling voxel size/bin width. Overall, radiomic features from T2 maps have proven to be less sensitive to image preprocessing than those from T1 maps, especially when varying bin width. Our results might corroborate the potential of radiomics from T1/T2 mapping in HCM and hopefully in other myocardial diseases.

## Introduction

Hypertrophic cardiomyopathy (HCM) is a genetically determined disease that affects about 1:500 people in the general adult population^1^. It is characterized by left ventricular (LV) hypertrophy, myofibrillar disarray, and myocardial fibrosis. Cardiac magnetic resonance (CMR) imaging has an important role in diagnosis, risk stratification, and treatment planning in HCM and, according to the European Society of Cardiology guidelines, it should be performed, at least as an initial evaluation, for all HCM patients^2^.

Actually, CMR imaging is considered the gold standard for the evaluation of ventricular morphology and function, as well as for the assessment of ventricular wall thickness^3,4^. Furthermore, late gadolinium enhancement (LGE) imaging enables the identification of focal myocardial fibrosis, which has been demonstrated to have a negative prognostic value in HCM patients^3,4^. More recently, cardiac T1 and T2 mapping techniques have been employed to obtain a more quantitative evaluation of myocardial tissue characteristics. Previous studies have consistently found that T1 and T2 values, as well as extracellular volume (ECV) values obtained from pre- and post-contrast T1 maps, are increased in HCM patients and have a potential prognostic role^5–11^. Actually, both native (i.e., without contrast administration) T1 and ECV values are considered markers of myocardial fibrosis^12^. On the other hand, T2 mapping is currently considered the gold standard for the evaluation of myocardial edema. Increased T2 values, probably related to low grade myocardial inflammation and dilated lymphatic channels^13,14^, have been demonstrated in HCM patients and have been proven to be associated with an increased arrhythmic risk^15,16^, as well as with more severe myocardial injury^17^. However, the usefulness of these mapping techniques is limited by a partial overlap of T1 and T2 values between HCM patients and healthy controls, as well as between HCM patients and patients with other myocardial diseases^18–21^.

Radiomics is a novel tool that involves the extraction of a large number of quantitative morphological and textural characteristics (i.e., radiomic features) from digital medical images^22^. The underlying idea is that medical images are data containing objective and quantitative information, which is not obtainable from qualitative visual inspection as usually performed in routine clinical practice. In addition to other available data from demographics, pathology, blood biomarkers, and genomics, radiomic features can be used for diagnostic, prognostic or predictive purposes exploiting statistical or machine learning (ML) methods^22–24^.

While radiomic analysis has been mainly applied in the field of oncology, there is a growing interest in improving the diagnostic accuracy and prognostic value of CMR imaging by exploiting radiomic techniques^25–30^. Recently, radiomics has aroused as a useful tool for unveiling myocardial tissue characteristics in HCM patients^18–21,31–36^. In this regard, previous studies focused mainly on traditional T1-weighted, T2-weighted, cine, and LGE images^18,31–35^. However, given their inherent quantitative nature, T1 and T2 maps might be particularly suitable for radiomic analysis. So far, only a few studies have applied texture analysis to T1 maps in patients with HCM^19–21,36^. They have found texture features able to discriminate among hypertensive heart disease (HHD), HCM, and healthy subjects^19,21^, between HCM patients with different genetic mutations^20^, as well as between LGE+ and LGE− patients^36^.

Despite the increasing interest in radiomics of CMR imaging, its proper application deserves some caution and a preliminary assessment of possible radiomic features dependence on various factors. Indeed, each step of the radiomic workflow (i.e., image acquisition and reconstruction, image segmentation, image preprocessing, image filtering, and feature extraction) could influence features estimation, thus potentially affecting their discriminative or predictive power^37,38^. Several previous studies have assessed the sensitivity of radiomic features estimation to various elements in computed tomography (CT)^39–41^ and nuclear medicine (NM)^42,43^ imaging, as well as in magnetic resonance imaging (MRI), for various applications^44–55^. On the other hand, only three studies have assessed some of these issues for CMR imaging^56–58^. In particular, Jang et al.^56^ have investigated both test–retest repeatability, as well as inter- and intra-observer reproducibility, in phantoms, healthy participants, and patients who were referred for clinical CMR imaging. They have observed different repeatability patterns among acquisition sequences in in vivo test–retest studies. Moreover, in an inter- and intra-observer reproducibility analysis, 32–47% and 61–73% out of 1023 features, respectively, were identified as reproducible, showing intraclass correlation coefficient (ICC) ≥ 0.8. Across all analyses, first order and gray level co-occurrence matrix were the most frequently identified reproducible radiomic feature classes. Another CMR imaging study by Jang et al.^57^ has evaluated, in a group of healthy participants and in a heterogeneous group of patients, the sensitivity of radiomic features estimate to changes in acquisition sequence parameters including flip angle, in-plane spatial resolution, slice thickness, and parallel imaging technique. As a whole, they have found that approximately 60% of the 4007 considered radiomic features were robust to changes in any acquisition parameters (i.e., standardized Cohen’s mean difference < 0.2), with qualitative acquisition sequences (i.e., cine balanced steady-state free-precession, T1-weighted spoiled gradient-echo, T2-weighted turbo spin-echo) and quantitative T1/T2 mapping most sensitive to changes in in-plane spatial resolution and flip angle, respectively. Alis et al.^58^ have evaluated the inter-observer reproducibility of radiomic features, as well as the influence of cardiac phases (i.e., systole, diastole or a 4D stack of cine images covering the cardiac cycle) on radiomic features estimation, from non-enhanced cine CMR images of healthy subjects. For the inter-observer analysis, they have found that the number of radiomic features with ICC > 0.8/0.85/0.9 changed with different phases of the cardiac cycle, and that texture features belonging to the gray level size zone matrix showed the worst performance. Moreover, approximately 55% out of 352 radiomic features showed a variability (in terms of coefficient of variation) > 20% through the cardiac cycle, with gray level dependence matrix and gray level run length matrix classes demonstrating less variability compared with other feature classes.

Image resampling and discretization, as well as spatial filtering, are some steps of the radiomic workflow that may be performed as preprocessing before the radiomic features extraction from the acquired image data^59,60^. In particular, image interpolation at the same voxel size is a common and recommended practice (especially in retrospective studies) to reduce any heterogeneity in acquisition voxel size, while image discretization is required to make texture feature estimation computationally less burdensome^42,59^. Moreover, the application of spatial filters before radiomic features estimation could allow uncovering further tissue characteristics. Notably, such a preprocessing actually alters acquired image data and possibly their radiomic characteristics. Nonetheless, so far, no work has specifically assessed the impact of both image resampling/discretization and spatial filtering on the estimate of radiomic features from CMR imaging. Therefore, this work aimed to comprehensively assess, for the first time, the effect of preprocessing—in terms of voxel size resampling, map values discretization, and spatial filtering—on radiomic features estimated from CMR T1 and T2 mapping in a group of HCM patients.

## Methods

### Subjects

We retrospectively identified 26 subjects (12 females, 14 males), referred for clinical CMR imaging between November 2013 and July 2020 for known or suspected HCM, for whom a comprehensive MRI study including both T1 and T2 mapping sequences was performed. The HCM diagnoses followed the latest European Society of Cardiology guidelines and were based on the presence of LV wall thickness ≥ 15 mm in one or more myocardial segments, not explained solely by loading conditions^2^. Clinical characteristics of the patient group are detailed in Table 1. The study was approved by the local ethics committee of the Azienda USL Toscana Nord Ovest (Pisa, Italy). Written informed consent was obtained from all subjects, and all experiments were performed in accordance with relevant guidelines and regulations.

**Table 1:** Clinical and CMR-derived chacteristics (mean ± standard deviation) of 26 patients with HCM.

**Clinical and CMR-derived characteristics**	
Age (years)	66 ± 11
LV ED volume (ml/m^2^)	74 ± 15
LV ES volume (ml/m^2^)	23 ± 14
LV stroke volume (ml/m^2^)	51 ± 13
LV ejection fraction (%)	70 ± 15
Mass (g/m^2^)	96 ± 21
RV ED volume (ml/m^2^)	61 ± 13
RV ES volume (ml/m^2^)	23 ± 6
RV stroke volume (ml/m^2^)	38 ± 9
RV ejection fraction (%)	63 ± 6
Thickness (mm)	19 ± 3
LGE	19/26

*LV* left ventricular, *ED* end diastolic, *ES* end systolic, *RV* right ventricular, *LGE* late gadolinium enhancement.

### CMR imaging

All CMR imaging examinations were performed using a 1.5 T MRI scanner system (MAGNETOM Avanto, Siemens Healthcare, Erlangen, Germany) equipped with 45 mT/m gradients strength and a 12-channel phased array coil.

Cine images were performed in the 2- and 4-chamber view planes (3 slices each) and in short-axis view (8–14 slices encompassing the entire LV), using a TrueFISP sequence (TR = 2.5 ms, TE = 1.2 ms, slice thickness = 8 mm).

Both T1 and T2 maps were obtained in the short axis view (a single slice located where myocardial thickness, evaluated with cine images, was maximum). T1 mapping was performed utilizing a modified look-locker inversion recovery (MOLLI) pulse sequence with a 3–3–5 acquisition scheme^61^. Pulse sequence parameters were as follows: TE/TR = 1.14/2.5 ms, flip angle = 35°, matrix size = 126 × 192, in-plane resolution ranged from 1.77 mm × 1.77 mm to 2.34 mm × 2.34 mm, typical field of view = 380 mm × 275 mm, slice thickness = 8 mm. T2 maps were obtained utilizing a T2-prepared TrueFISP sequence^62^ with the following parameters: T2 preparation time = 0/24/55 ms, TR = 4 × *R*–*R*, flip angle = 70°, matrix size = 126 × 192, in-plane resolution ranged from 1.77 × 1.77 mm to 2.34 × 2.34 mm, typical field of view = 380 mm × 275 mm, slice thickness = 6 mm.

Then, 10–15 min after intravenous administration of 0.2 mmol/kg of gadolinium DTPA (Magnevist, Schering), gadoteric acid (Dotarem, Guerbet) or gadoteridol (Prohance, Bracco), LGE images were acquired in 2- and 4-chamber view of the LV (3 slices each), as well as in the short-axis view (8–14 slices encompassing the entire LV) using a 2D phase-sensitive inversion recovery (PSIR) sequence (TR = 700 ms, TE = 1.09 ms, slice thickness = 8 mm, inversion time = 200–300 ms, typical in-plane resolution = 2.4 mm × 3.2 mm).

### Preprocessing of T1 and T2 maps

For each subject, a region of interest (ROI) covering the entire myocardium was manually segmented by a single expert radiologist in cardiac MRI, with 15 years of experience, using 3D Slicer (Version 4.11.2)^63,64^. ROIs were delineated on T1 and T2 maps separately, avoiding voxels with potential partial volume effect. Supplementary Figure S1 shows an example myocardium ROI for a representative HCM patient.

Given that T1 and T2 maps allowed acquiring only a single slice, resampling voxel size was performed through 2D interpolation by using the B-spline interpolation algorithm (with the origins of interpolation and original image grids aligned together^59^). The original in-plane spatial resolution ranged from 1.77 mm × 1.77 mm to 2.34 mm × 2.34 mm across patients for both T1 and T2 maps. Therefore, T1 and T2 maps were resampled to an in-plane isotropic spatial resolution of 1.8 mm, 1.9 mm, 2.0 mm, 2.1 mm, 2.2 mm, 2.3 mm, and 2.4 mm. Resampling voxel size was performed for different bin widths (see below).

T1 and T2 maps discretization was carried out following a fixed bin width approach, as recommended by the Image Biomarker Standardisation Initiative (IBSI) when dealing with quantitative data^59^. In particular, for each resampling voxel size (i.e., 1.8, 1.9, 2, 2.1, 2.2, 2.3, and 2.4 mm in-plane isotropic spatial resolution), bin width values of 3.60 ms, 3.95 ms, 4.30 ms, 4.65 ms, 5.00 ms, 5.35 ms, 5.70 ms, 6.05 ms, and 6.40 ms were employed for T1 maps, while bin width values of 0.49 ms, 0.50 ms, 0.51 ms, 0.52 ms, 0.53 ms, 0.54 ms, 0.55 ms, 0.56 ms, and 0.57 ms were employed for T2 maps. Indeed, given that the median (across HCM patients) range of T1 and T2 values (i.e., the difference between the maximum and minimum T1 and T2 values of voxels within the ROI) was 265 ms and 26 ms, respectively, bin width values were chosen in such a way that the number of quantization levels of T1 and T2 maps was within the range of 30–130, for each HCM patient. This approach, adopted also in previous technical studies^40,41,52,54^, has the potential to limit variability in radiomic features estimation^43,65,66^.

Different spatial filters were applied on T1 and T2 maps: 2D wavelets (Daubechies 3) yielding four filtered maps (i.e., wavelet-LH, -HL, -HH, -LL, where L/H refers to the combination of low-/high-pass filters applied in the horizontal and vertical direction), gradient magnitude of the map (i.e., gradient filter), the square of the map values (i.e., square filter), and the square root of the absolute map value (i.e., square-root filter). Specifically, in order to restrict the large number of possible combinations, image filtering was performed at fixed resampling in-plane isotropic spatial resolution of 2.1 mm, and at fixed bin width of 6 ms and 0.56 ms for T1 and T2 maps, respectively, yielding median (across subjects) numbers of quantization levels between 30 and 130^43,65,66^.

All preprocessing steps (i.e., T1/T2 maps resampling, discretization, and spatial filtering) and subsequent radiomic features estimation were carried out by using the open source PyRadiomics library^67^ (Version 3.0.1) with Python (Version 3.7.3), running on a MacBook Air (macOS Version 10.14) with a 1.8 GHz Intel Core i5 CPU.

### Radiomic features estimation

Given that the used acquisition sequences allowed obtaining T1 and T2 maps on a single slice, the 2D versions of radiomic features were considered. For each ROI and preprocessing combination, in terms of resampling voxel size and bin width, a total of 98 features were obtained: 9 2D-shape features, 16 first order features (14 intensity-based statistical features and 2 intensity histogram features, namely Entropy and Uniformity), and 73 second order features (i.e., textural features) from gray level co-occurrence matrix (GLCM, 22 features), gray level run length matrix (GLRLM, 16 features), gray level size zone matrix (GLSZM, 16 features), gray level dependence matrix (GLDM, 14 features, with coarseness parameter α = 0), and neighborhood gray tone difference matrix (NGTDM, 5 features). Second order features estimation was performed according to the Chebyshev norm with a distance of 1 pixel. GLCM and GLRLM features were computed from each 2D directional matrix (i.e., at 0°, 45°, 90°, and 135°) and averaged over 2D directions.

For each ROI and spatial filter applied on T1 and T2 maps, a total of 89 features were estimated, i.e. all the above features but the shape features. Indeed, given that shape features are usually estimated regardless of the applied image filter, they were not included in our analysis.

All radiomic features were computed in accordance with the definitions provided by the IBSI, with shape features computed in 2D instead of the proposed 3D version. It is worth noting that the first order feature of Kurtosis calculated by PyRadiomics was in accordance with the IBSI except for an offset value (i.e., 3).

### Statistical analysis

In this study, three different effects on radiomic features estimation were assessed for T1 and T2 maps singularly:

A. For each bin width, the effect of using different resampling voxel sizes.
B. For each voxel size, the effect of using different bin widths.
C. At fixed resampling voxel size and bin width, the effect of using different spatial filters.

For each effect of interest, any variability in radiomic features estimate was assessed through ICC analysis^68,69^. In particular, the two-way mixed effects model, with single rater and absolute agreement, was selected. Accordingly, the ICC coefficient was calculated as:

1 $$ICC = \frac{{MS_{R} - MS_{E} }}{{MS_{R} + (k - 1)MS_{E} + \frac{k}{n}(MS_{C} - MS_{E} )}}.$$

where MS_R_ = mean square for rows (i.e., between subjects), MS_E_ = mean square for error, MS_C_ = mean square for columns (i.e., between measurements), n = number of subjects and k = number of raters, with ICC matrices realized considering each resampling voxel size, quantization bin width or image filter as a rater. ICC values range between 0 (maximum variability) and 1 (minimum variability) and express the variability of radiomic features estimate associated with the effect of interest with respect to the variance between subjects (i.e., a relative variability). Radiomic features were stratified based on their degree of relative variability^37,52^: high (ICC ≤ 0.5), considerable (0.5 < ICC ≤ 0.75), moderate (0.75 < ICC ≤ 0.9), and low (0.9 < ICC ≤ 1) relative variability.

In order to characterize in greater detail and absolutely the variability in radiomic feature estimate, an additional analysis of the coefficient of variation (CV) was performed. In particular, CVs were computed as the percentage ratio between standard deviation and absolute mean values of features estimate obtained by varying one element of interest (resampling voxel size, bin width or spatial filter) while fixing the others. Accordingly, this yielded 26 × 9 CVs for effect A (i.e., 26 subjects and 9 fixed bin widths), 26 × 7 CVs for effect B (i.e., 26 subjects and 7 fixed resampling voxel size), and 26 CVs for effect C (i.e., 26 subjects with fixed resampling voxel size and bin width).

Any linear correlation between radiomic features estimate and resampling voxel size or bin width was assessed by a repeated measures correlation analysis, namely rmcorr^70^. This technique accounts for non-independence among observations (i.e., repeated measures of the same radiomic features of the same subject by varying one preprocessing element) using analysis of covariance (ANCOVA) to statistically adjust for the variability between subjects. A p-value < 0.05, adjusted for multiple comparisons using Bonferroni correction, was considered significant.

Statistical analysis was carried out by using R (Version 3.6.2) software package in the RStudio (Version 1.2.5033) environment^71^.

## Results

The entire time taken by the PyRadiomics tool to extract all the combinations of radiomic features for effects A or B was approximately 35 s, while the estimation process for effect C took approximately 10 s.

**Figure 1:**
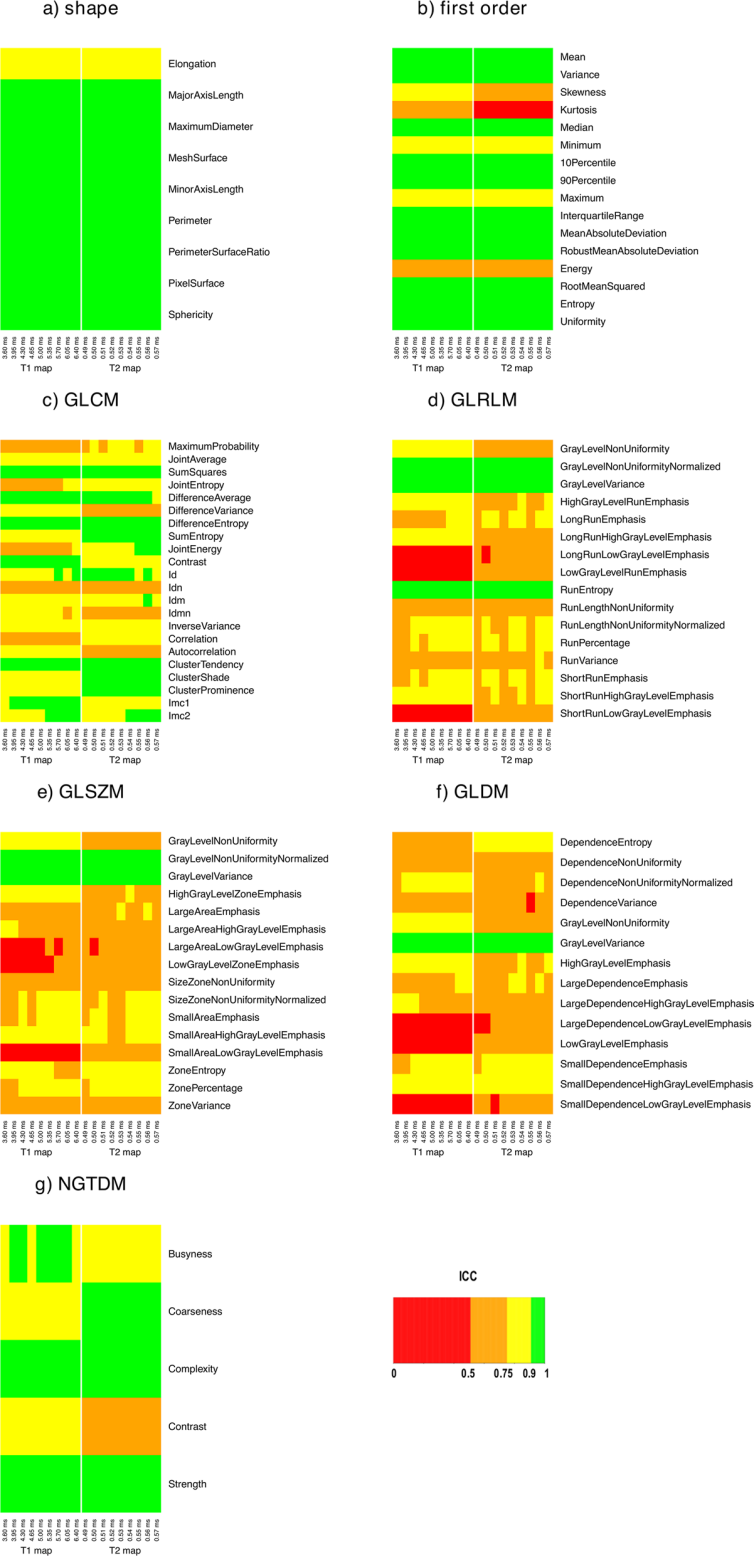
ICC results for effect A, for both T1 and T2 maps. The heatmap of each radiomic features class (i.e., shape, first order, GLCM, GLRLM, GLSZM, GLDM, and NGTDM) shows the degree of relative variability in radiomic features estimate when using different resampling voxel sizes for different bin widths (i.e., 3.60, 3.95, 4.30, 4.65, 5.00, 5.35, 5.70, 6.05, and 6.40 ms for T1 maps and 0.49, 0.50, 0.51, 0.52, 0.53, 0.54, 0.55, 0.56, and 0.57 ms for T2 maps).

**Figure 2:**
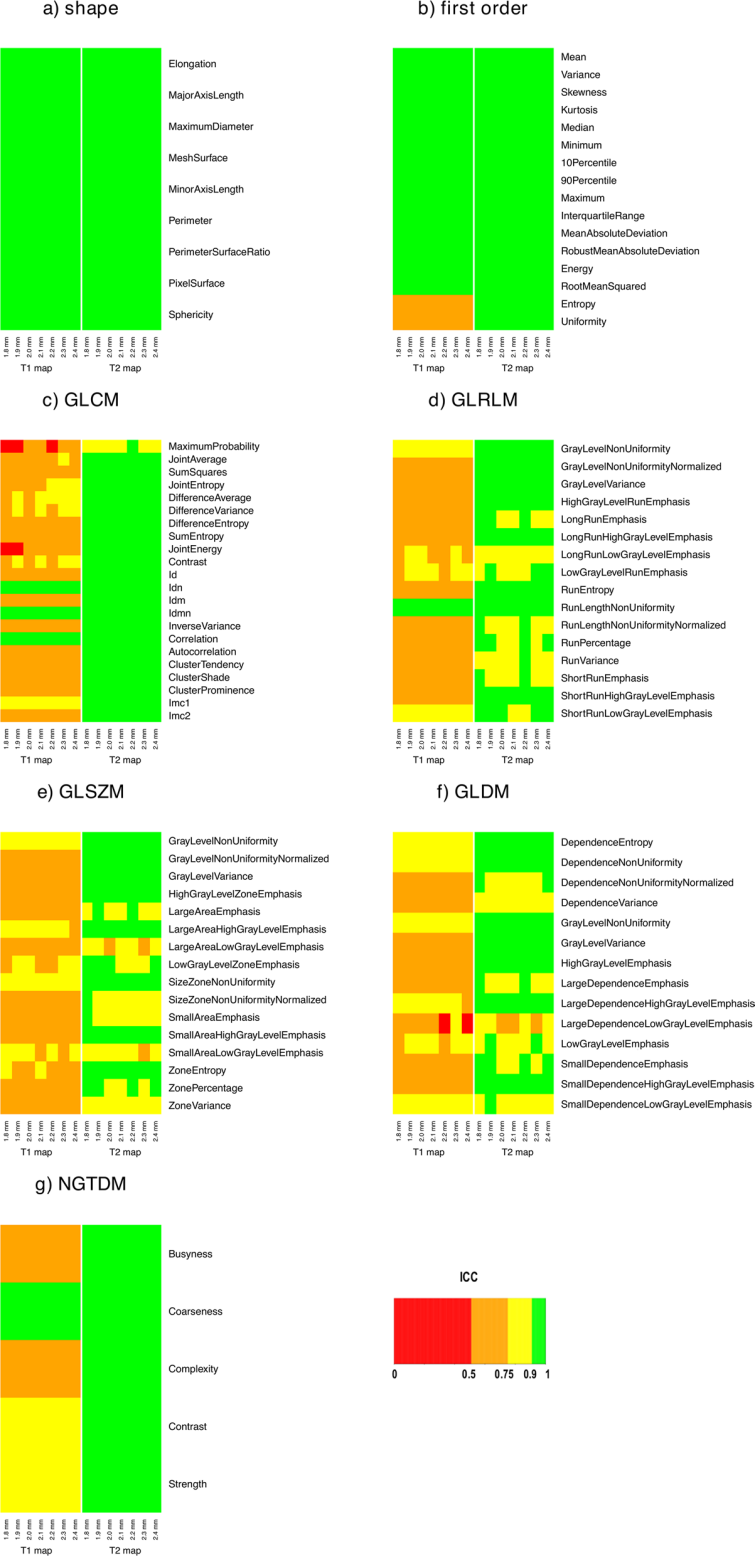
ICC results for effect B, for both T1 and T2 maps. The heatmap of each radiomic features class (i.e., shape, first order, GLCM, GLRLM, GLSZM, GLDM, and NGTDM) shows the degree of relative variability in radiomic features estimate when using different bin widths for different resampling voxel sizes (i.e., 1.8, 1.9, 2.0, 2.1, 2.2, 2.3, and 2.4 mm for both T1 and T2 maps).

ICC results for the effects of interest A and B are reported in Figs. 1 and 2, respectively, for both T1 and T2 maps. ICC values were > 0.9 for radiomic features belonging to the shape class. In general, first and second order features estimated from T2 maps showed a lower relative variability than those estimated from T1 maps, especially for effect B where ICC values were always in moderate or low ranges (with few exceptions for some resampling voxel size values, i.e., GLSZM-LargeAreaLowGrayLevelEmphasis, GLSZM-SmallAreaLowGrayLevelEmphasis, and GLDM-LargeDependenceLowGrayLevelEmphasis). For both T1 and T2 maps, effect C resulted in higher relative variability (ICC ≤ 0.5) of radiomic features estimate as compared to effect A and B (see Supplementary Fig. S2).

**Figure 3:**
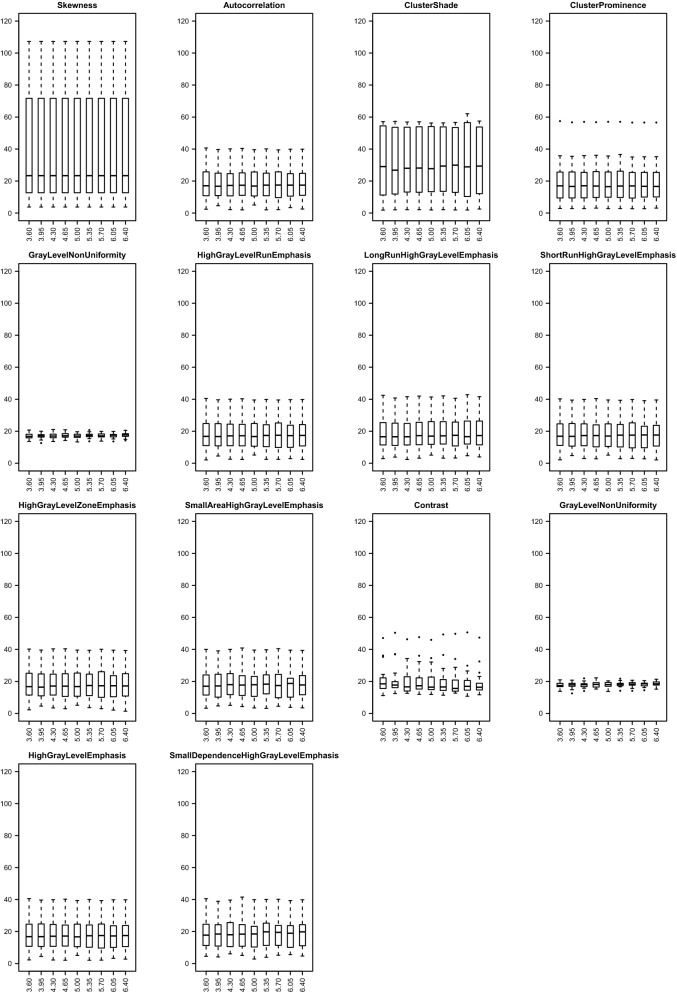
Box and whisker plots of CV (%) values of enrolled subjects for effect A assessed on T1 maps. Radiomic features with both median (across subjects) CV > 15% and ICC > 0.75 for each bin width (i.e., 3.60, 3.95, 4.30, 4.65, 5.00, 5.35, 5.70, 6.05, and 6.40 ms) are shown.

**Figure 4:**
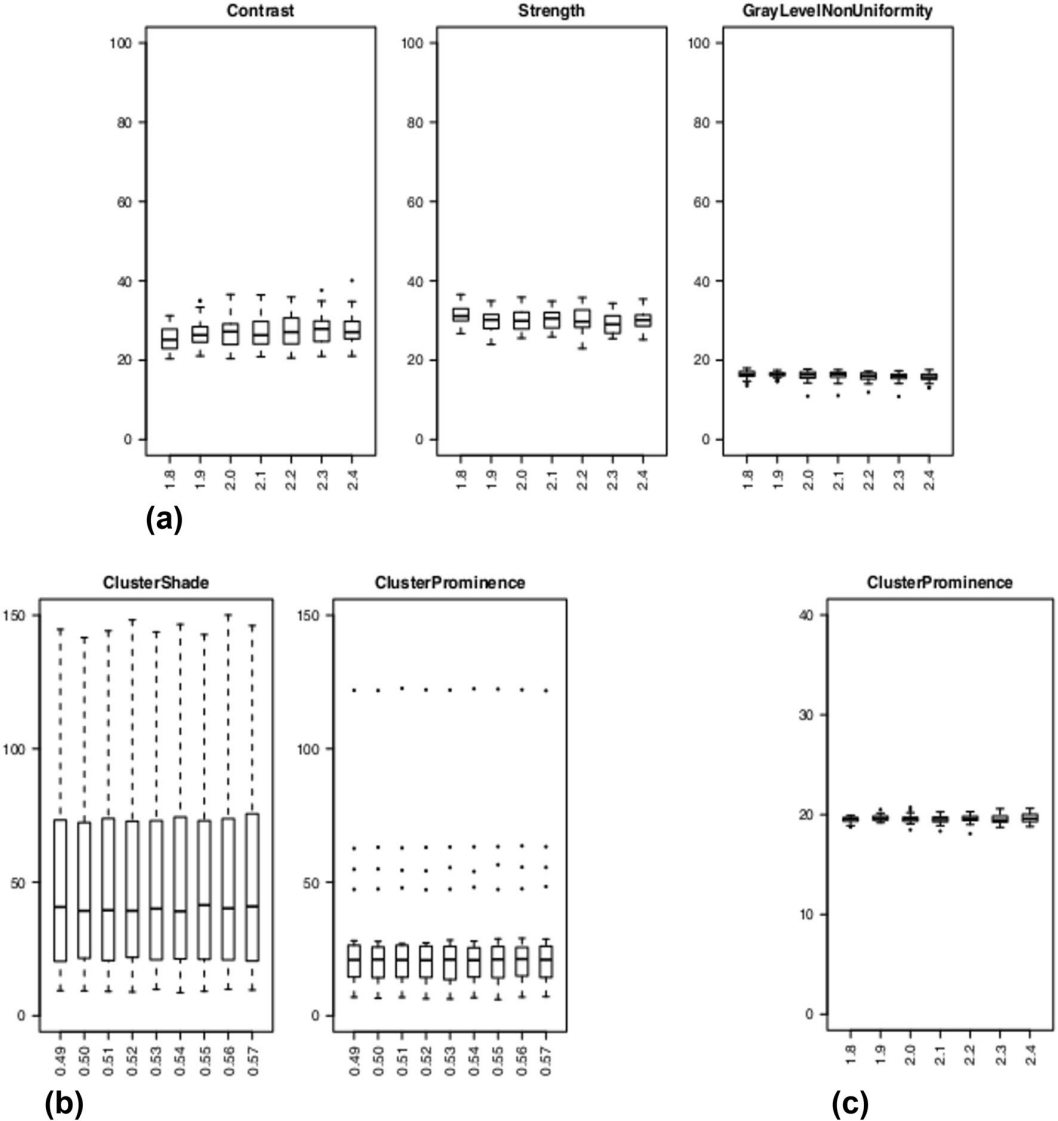
Box and whisker plots of CV (%) values of enrolled subjects for effect B assessed on T1 maps **(a)** and for effect A **(b)**/B **(c)** assessed on T2 maps. **(a,c)** Radiomic features with both median (across subjects) CV > 15% and ICC > 0.75 for each resampling voxel size (i.e., 1.8, 1.9, 2.0, 2.1, 2.2, 2.3, and 2.4) are shown for T1 **(a)** and T2 maps **(c)**. **(b)** Radiomic features computed from T2 maps with both median (across subjects) CV > 15% and ICC > 0.75 for each bin width (i.e., 0.49, 0.50, 0.51, 0.52, 0.53, 0.54, 0.55, 0.56, and 0.57 ms) are shown.

For effects A and B, Table 2 summarizes the median (across subjects and bin widths/voxel sizes for effect A/B) CV values of radiomic features estimated from T1 and T2 maps. The CV was less than 40% for both effects as well as for T1 and T2 (with few exceptions for effect B assessed on T1 maps, i.e., GLCM-ClusterShade, GLCM-ClusterProminence, NGTDM-Complexity, and GLDM-SmallDependenceHighGrayLevelEmphasis). Specifically, for effect B assessed on T2 maps, this absolute variability was < 20%. Moreover, Figs. 3, 4a–c show radiomic features with both median (across subjects and bin widths/voxel sizes for effect A/B) CV > 15% and ICC > 0.75 for effect A/B assessed on T1 and T2 maps, respectively. For effect C, median (across subjects) CV values of radiomic features estimated from T1 and T2 maps are summarized in Supplementary Table S1, showing that the absolute variability associated with this effect was markedly greater than that associated with effects A and B.

**Table 2:** Median CV (%) of radiomic features estimate for effect A and B, for both T1 and T2 maps. The median CV was calculated across subjects and bin widths/voxel sizes for effect A/B.

	A (T1)	B (T1)	A (T2)	B (T2)		A (T1)	B (T1)	A (T2)	B (T2)
**Shape**					**GLRLM**				
Elongation	1.51	0.00	1.65	0.00	GrayLevelNonUniformity	17.23	14.81	18.14	4.28
MajorAxisLength	0.99	0.00	0.82	0.00	GrayLevelNonUniformityNormalized	3.32	16.00	2.77	4.55
MaximumDiameter	0.94	0.00	0.84	0.00	GrayLevelVariance	7.31	37.55	7.20	9.70
MeshSurface	2.67	0.00	2.35	0.00	HighGrayLevelRunEmphasis	17.16	36.92	10.12	9.55
MinorAxisLength	1.03	0.00	0.90	0.00	LongRunEmphasis	2.54	3.39	1.99	1.27
Perimeter	1.27	0.00	0.96	0.00	LongRunHighGrayLevelEmphasis	16.95	33.68	10.52	8.88
PerimeterSurfaceRatio	2.55	0.00	2.39	0.00	LongRunLowGrayLevelEmphasis	26.94	17.67	25.70	8.58
PixelSurface	2.66	0.00	2.36	0.00	LowGrayLevelRunEmphasis	23.29	15.45	22.44	7.68
Sphericity	1.71	0.00	1.51	0.00	RunEntropy	1.31	3.49	1.16	1.04
**First order**					RunLengthNonUniformity	17.61	3.25	18.48	1.24
Mean	0.17	0.00	0.23	0.00	RunLengthNonUniformityNormalized	1.53	2.12	1.28	0.83
Variance	7.07	0.00	7.10	0.00	RunPercentage	0.83	1.13	0.68	0.42
Skewness	23.38	0.00	36.34	0.00	RunVariance	17.10	22.36	14.31	8.65
Kurtosis	6.25	0.00	15.42	0.00	ShortRunEmphasis	0.61	0.85	0.51	0.33
Median	0.14	0.00	0.19	0.00	ShortRunHighGrayLevelEmphasis	17.31	37.67	10.18	9.74
Minimum	2.18	0.00	1.28	0.00	ShortRunLowGrayLevelEmphasis	22.61	14.62	22.31	7.55
10Percentile	0.33	0.00	0.26	0.00	**GLSZM**				
90Percentile	0.26	0.00	0.57	0.00	GrayLevelNonUniformity	15.14	11.14	17.05	3.74
Maximum	1.56	0.00	4.08	0.00	GrayLevelNonUniformityNormalized	3.98	15.26	3.20	4.56
InterquartileRange	3.39	0.00	3.32	0.00	GrayLevelVariance	7.64	36.63	7.60	9.51
MeanAbsoluteDeviation	3.16	0.00	2.50	0.00	HighGrayLevelZoneEmphasis	16.89	36.87	9.77	9.47
RobustMeanAbsoluteDeviation	2.61	0.00	2.45	0.00	LargeAreaEmphasis	10.03	14.02	8.17	5.17
Energy	19.05	0.00	19.44	0.00	LargeAreaHighGrayLevelEmphasis	21.57	24.31	13.95	7.79
RootMeanSquared	0.17	0.00	0.24	0.00	LargeAreaLowGrayLevelEmphasis	29.79	26.74	29.44	11.17
Entropy	0.93	4.70	0.88	1.36	LowGrayLevelZoneEmphasis	21.64	15.94	20.50	6.43
Uniformity	3.22	16.23	2.78	4.65	SizeZoneNonUniformity	14.60	10.78	16.61	4.46
**GLCM**					SizeZoneNonUniformityNormalized	4.86	6.38	4.19	2.82
MaximumProbability	12.90	18.94	11.52	8.92	SmallAreaEmphasis	2.19	2.93	1.90	1.26
JointAverage	9.00	18.28	6.05	4.80	SmallAreaHighGrayLevelEmphasis	17.62	39.43	10.35	10.19
SumSquares	6.43	37.89	5.43	9.78	SmallAreaLowGrayLevelEmphasis	22.68	14.50	22.84	7.56
JointEntropy	2.01	2.48	1.78	0.84	ZoneEntropy	1.99	2.24	1.74	0.88
DifferenceAverage	7.19	18.81	6.32	4.90	ZonePercentage	3.25	4.48	2.67	1.75
DifferenceVariance	15.11	37.67	15.53	9.73	ZoneVariance	25.96	31.92	22.03	13.92
DifferenceEntropy	2.12	6.35	2.09	1.72	**GLDM**				
SumEntropy	1.33	3.70	1.13	1.12	DependenceEntropy	2.20	1.81	1.92	0.82
JointEnergy	9.57	16.96	8.26	5.66	DependenceNonUniformity	14.85	9.97	16.66	3.93
Contrast	14.07	37.79	13.24	9.77	DependenceNonUniformityNormalized	6.99	9.97	5.74	3.93
Id	4.44	10.53	3.90	2.92	DependenceVariance	19.39	21.59	15.42	9.81
Idn	0.76	0.05	0.97	0.06	GrayLevelNonUniformity	18.14	16.23	18.64	4.65
Idm	6.67	15.30	5.89	4.28	GrayLevelVariance	7.16	37.90	7.07	9.78
Idmn	0.26	0.02	0.32	0.02	HighGrayLevelEmphasis	17.22	36.96	10.38	9.60
InverseVariance	6.69	15.05	6.45	4.77	LargeDependenceEmphasis	10.01	13.61	8.18	5.31
Correlation	5.44	0.15	7.95	0.25	LargeDependenceHighGrayLevelEmphasis	22.07	24.58	14.42	7.93
Autocorrelation	17.22	36.95	11.00	9.62	LargeDependenceLowGrayLevelEmphasis	35.67	28.91	33.90	12.69
ClusterTendency	7.43	37.89	6.33	9.79	LowGrayLevelEmphasis	24.40	15.39	23.19	7.65
ClusterShade	28.91	56.94	39.95	14.85	SmallDependenceEmphasis	4.34	5.97	3.60	2.39
ClusterProminence	16.89	75.29	20.99	19.57	SmallDependenceHighGrayLevelEmphasis	19.39	42.05	11.23	10.99
Imc1	6.10	8.35	7.34	3.16	SmallDependenceLowGrayLevelEmphasis	21.93	10.94	22.51	6.38
Imc2	0.22	0.57	0.65	0.41					
**NGTDM**									
Busyness	15.81	28.10	12.41	8.34					
Coarseness	9.25	2.84	10.35	1.21					
Complexity	12.00	49.44	11.93	12.96					
Contrast	16.93	26.73	17.60	7.60					
Strength	11.37	30.21	14.18	8.08

The results of the analysis of the linear correlation between radiomic features estimate and voxel size/bin width are reported in Supplementary Tables S2–S5 for T1 and T2 maps, respectively. Most of the textural features showed a significant linear correlation with voxel size and bin width within the considered range of variation, with some exceptions (see Supplementary Tables S4) among radiomic features estimated from T2 maps when varying resampling voxel size (i.e., 6 GLCM, 7 GLRLM, 6 GLSZM, 6 GLDM, and 3 NGTDM radiomic features).

## Discussion

Recently, some studies have applied radiomic analysis to CMR imaging in order to assess whether this tool could reveal myocardial phenotypic alterations in HCM patients^18–21,31–36^. By using conventional CMR imaging (i.e., T1-weighted, T2-weighted STIR, LGE, and cine images), radiomic analysis has allowed discriminating between HCM patients and healthy controls^18,31^, as well as distinguishing among different aetiologies of left ventricular hypertrophy, such as HCM, HHD, cardiac amyloid, and aortic stenosis^32^. Moreover, some studies have focused on the prognostic role of radiomic analysis of LGE images in HCM, suggesting that texture features are related to the arrhythmic risk^33,35^ and adverse clinical outcome^34^. Notably, only a few pilot HCM studies^19–21,36^ have applied radiomic analysis to T1 maps. Specifically, two studies^19,21^ have focused on the diagnostic capability of radiomic analysis to discriminate between healthy controls and HCM/HHD patients. Also, Neisius et al.^36^, have found that radiomic analysis applied to T1 maps was able to distinguish between LGE+ and LGE–, identifying a subgroup of subjects in whom gadolinium administration could be safely avoided. Wang et al.^20^ have found that radiomic analysis of T1 maps can discriminate HCM patients with different mutations of sarcomere-related genes.

Previous studies have investigated the effect of image preprocessing on radiomic features estimation as a function of various factors, which include imaging technique/modality and anatomical region^40–43,47,48,51–55,72^. Nonetheless, to the best of our knowledge, this is the first study aimed at assessing the effect of both image resampling/discretization and spatial filtering on radiomic features estimated from CMR T1 and T2 maps. Thus, in a group of HCM patients, we performed a relatively comprehensive analysis, which considered multiple elements such as voxel size resampling, T1/T2 values discretization, and spatial filtering.

In radiomic studies, voxel size resampling is a recommended and commonly employed preprocessing step when analyzing data with different acquisition protocols or from different scanners, which can result in different acquisition voxel sizes^59^. For both T1 and T2 maps, we found that textural features belonging to GLCM and NGTDM classes showed moderate relative variability (0.75 < ICC ≤ 0.9) when varying resampling voxel size, except for a limited number of radiomic features (Fig. 1). Conversely, the estimate of textural features of GLRLM, GLSZM, and GLDM classes showed a higher relative variability when varying resampling voxel size, with few exceptions (e.g., GrayLevelVariance from GLRLM, GLSZM, and GLDM, which presented ICC > 0.9 for both T1 and T2 maps). In general, radiomic features belonging to shape and first order classes were characterized by higher ICC values than textural features (Fig. 1), indicating that resampling voxel size has less impact on their estimate. In particular, shape radiomic features had ICC values > 0.9 (Elongation was the only exception, showing 0.75 < ICC ≤ 0.9) and median CV values within 0.8%-2.7% (Table 2) for both T1 and T2 maps. Most of first order radiomic features presented ICC values > 0.75, with only few features showing considerable (i.e., Energy from T1 and T2 maps, Kurtosis from T1 maps, and Skewness from T2 maps) or high (i.e., Kurtosis from T2 maps) relative variability. Furthermore, we revealed that even some radiomic features from T1 and T2 maps with ICC > 0.75 can have a non-negligible absolute variability in terms of CV (up to 30% or more, for single subjects) when varying resampling voxel size. In this regard, we found that T1 maps presented an appreciably higher number of radiomic features with ICC > 0.75 and median CV > 15% than T2 maps (Figs. 3, 4b). For most textural features, a significant linear correlation between their estimates and resampling voxel sizes was observed (Supplementary Tables S2, S4).

Image discretization is another important step before radiomic features estimation, which allows simplifying rather complex computational operations. Given that T1 and T2 represent quantitative physical properties of tissues, we chose to apply a fixed bin width approach^59^. Bin width ranges of variation (i.e., 3.6–6.4 ms and 0.49–0.57 ms for T1 and T2 maps, respectively) were selected using the same criterion (i.e., median number of quantization levels between 30 and 130^43,65,66^), allowing a comparison between the results of T1 and T2 maps. A remarkable difference between T1 and T2 maps in sensitivity of radiomic features estimation to discretization was found. In general, textural features estimated from T1 maps showed higher variability than their estimates from T2 maps (ICC > 0.75). Only a limited number of T1 maps textural features were characterized by ICC > 0.75 (Fig. 2) and some of them still had median CV > 15% (Fig. 4a). Overall, CV values were greater for T1 maps (up to 75%) than for T2 maps (less than 20%) (see Table 2). As expected, shape and intensity-based statistical features (i.e., all first order features but Entropy and Uniformity) yielded ICC = 1 and CV = 0%. Indeed, these radiomic features are estimated by PyRadiomics, according to the IBSI recommendation, prior to discretization^59^ and hence this preprocessing step does not affect their estimate. The repeated measures correlation analysis showed that all radiomic textural features (with few exceptions) have a significant linear correlation between their estimates and bin width (Supplementary Tables S3, S5).

Digital image filters can be applied before radiomic features extraction to detect and emphasize tissue characteristics different from those usually obtained from original (i.e., unfiltered) images. In this regard, the IBSI has proposed a new reference manual, in order to define and standardize the implementation of image filters in radiomics software^60^. Given that a spatial filter can actually modify (even in a substantial way) T1 and T2 maps, we observed a relevant sensitivity of both first order and textural features estimates to image filter, with ICC ≤ 0.5 (Supplementary Fig. S2). On the other hand, the entity of this effect can vary with radiomic features and is not easily predictable especially for textural radiomic features. Indeed, as reported in Supplementary Table S1, median CVs of radiomic features estimate for different spatial filters can range from less than 10% to 200% or more.

The revealed effect of resampling voxel size and bin width on the estimate of myocardial radiomic features from T1 and T2 mapping, albeit limited in many cases, sustains the importance of reporting/describing in detail these aspects in clinical and research studies. In this regard, while previous studies involving myocardial T1 mapping have described the radiomic features extraction process quite exhaustively^19–21,36^, some information about image preprocessing was still missing. For instance, none of these studies have reported whether and how image discretization was performed (notably, Neisius et al.^19^ have specified the number of discretized intensity levels but only for the radiomic features belonging to the GLRLM class, while no discretization was indicated for the GLCM class). Furthermore, the studies conducted by Neisius et al.^19^ and Shi et al.^21^ have acquired images with different in-plane spatial resolutions (i.e., 2.1 and 1.3 mm, respectively) and employed slightly different preprocessing steps (e.g., range re-segmentation and intensity outlier filtering, respectively^59^). This could partly explain the obtained different results in terms of discriminative radiomic features and accuracy.

So far, previous HCM radiomic studies^19–21,36^ have exploited only T1 mapping, mainly because of its capability of revealing myocardial fibrosis. However, T2 maps are considered the gold standard for the evaluation of myocardial edema, which is a well-known negative prognostic factor in HCM^15,16^. Moreover, although T2 mapping is not per se capable of evaluating myocardial fibrosis, texture analyses might theoretically overcome this limitation, unveiling myocardial structural heterogeneity due to myofibrillar disarray and fibrosis^18,27^. Therefore, given also that, overall, radiomic features from T2 maps have proven to be characterized by a lower sensitivity to image resampling and discretization than radiomic features from T1 maps (especially when varying bin width), radiomics from T2 mapping might be exploited to obtain a complementary characterization of HCM, albeit this lower sensitivity does not necessarily imply higher discriminative or predictive power. In addition, it should be noted that, for both T1 and T2 mapping, the sensitivity of several radiomic features to resampling voxel size and bin width resulted rather independent of bin width and resampling voxel size, respectively. Moreover, only a limited number of radiomic features showed high relative variability (ICC ≤ 0.5) associated with different resampling voxel size or bin width. Some radiomic features with ICC > 0.75 still presented high variability (> 30%) in terms of CV for single subjects, suggesting that such radiomic data from different centers should be compared or pooled with caution.

We recognize and submit the following potential limitations and implications, respectively, of our study. First, while we analyzed only single slice acquisitions of T1 and T2 mapping, in HCM patients, myocardial changes may involve also visually non-hypertrophied myocardial segments. Therefore, a whole-heart coverage might provide a more comprehensive evaluation of disease burden and increase the diagnostic performance of CMR imaging. However, for a global/diffuse disease, it is considered adequate to analyze a single ROI on mid-cavity short-axis map^73^. Furthermore, the main aim of our exquisitely technical study was to assess the effect of image resampling and discretization on radiomic features estimated from typical cardiac T1 and T2 maps. Accordingly, we focused on a single short-axis slice, located where myocardial thickness was maximum and myocardial changes were assumed to be more severe, obtaining hence minimum partial volume effects, which can greatly affect regions of thinner myocardial segments. Moreover, this is a retrospective study that has enrolled subjects referred for clinical/routine CMR imaging, which requires avoiding too long acquisition times especially for non-collaborative patients. Second, myocardial T1 and T2 maps can be affected by noise, as well as by possible fitting errors and patient motion. In this regard, while appropriate denoising filters applied on T1- and T2-weighted images can be used to improve the precision and accuracy of computed T1 and T2 maps^74,75^, we adopted no denoising method. Third, in this technical study involving HCM patients, no clinical/diagnostic results tailored to this disease were reported. In this regard, in order to avoid any potential confounding effects in our analysis, we preferred to enroll only a group of homogeneous subjects with the same pathology. Therefore, this preliminary technical radiomic study on T1 and T2 mapping can be helpful and prodromic for further clinical studies on HCM. Nonetheless, we feel our analysis framework could be applied to other cardiac populations likely obtaining similar findings, albeit that would need further experimental studies to be confirmed. Fourth, image resampling and discretization are only two of the various steps of the radiomic pipeline, albeit important and recommended by the IBSI guidelines^59^. The issue of standardization in radiomic studies can involve additional factors, such as scanner performances, acquisition protocols, acquisition sequence parameters, and data analysis methods. However, we feel the results of our study can represent a potentially helpful step toward the standardization of radiomics in cardiac magnetic resonance imaging. Indeed, image resampling and discretization modify de facto acquired image data and possibly their radiomic characteristics. Therefore, professionals and researchers executing or planning clinical or research studies should be aware of this critical aspect of radiomics in cardiac magnetic resonance imaging and its entity for both T1 and T2 mapping. As for the practical implications of this study, we note that radiomic techniques allow extracting hundreds of features. In clinical and research studies employing radiomics and artificial intelligence methods, even when enrolling numerous subjects, it is hence mandatory to apply appropriate methods of features selection, in order to avoid any possible bias due to overfitting and obtain reliable results. While supervised or unsupervised methods of features selection can be used^76^, a limited sensitivity to image resampling and discretization can represent an additional criterion for selecting features. This does not necessarily imply higher discriminative or predictive power in clinical or research studies, which might also depend on the specific effect size associated with selected features, but still can improve reproducibility of results and comparison/pooling of data from different centers.

## Conclusions

Toward an optimization and standardization in radiomic applications, any potential dependence of radiomic features on various aspect of the radiomic workflow should be possibly taken into account when planning a clinical or research study. Nowadays, there is a growing interest in exploiting radiomic techniques in various clinical applications. In particular, the study of myocardial diseases represents an emerging and promising field of application. In this HCM study, while we observed a sensitivity of myocardial radiomic features from T1 and T2 mapping to some conventional image preprocessing procedures such as image resampling/discretization, this effect was very remarkable only when considering image filtering. Indeed, the sensitivity of radiomic features to different resampling voxel sizes and bin widths was limited for many textural radiomic features, as well as for shape and first order radiomic features. The estimate of most textural radiomic features showed a linear significant correlation with resampling voxel size and bin width. In general, radiomic features from T1 mapping were more sensitive to image preprocessing than radiomic features from T2 mapping, especially when varying bin width. Overall, our results might corroborate the potential of radiomics from T1 and T2 mapping in HCM and hopefully in other myocardial diseases.

## Supplementary Information

Supplementary Information.

## Data Availability

The datasets generated during and/or analysed during the current study are available from the corresponding author on reasonable request.
