# Supplementary material for: Image resampling and discretization effect on the estimate of myocardial radiomic features from T1 and T2 mapping in hypertrophic cardiomyopathy
Source: Sci Rep. 2022 Jun 17;12:10186. doi: 10.1038/s41598-022-13937-0 (PMC9205876; doi:10.1038/s41598-022-13937-0)
Supplement: Supplementary file 1 — Supplementary Information. [file 41598_2022_13937_MOESM1_ESM.pdf]

Image resampling and discretization effect on the estimate of myocardial radiomic features from T1 and T2 mapping in hypertrophic cardiomyopathy: Supplementary Material

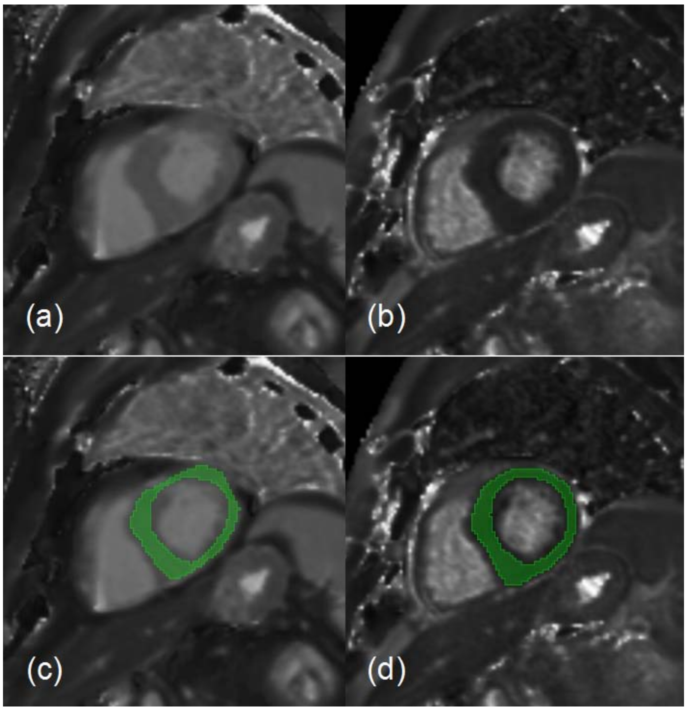

Supplementary Figure S1. T1 **(a)** and T2 **(b)** maps (short-axis view) of a representative HCM patient, with the corresponding manually segmented myocardium region of interest (ROI) showed in pane **(c)** and **(d)**, respectively. Myocardium ROI size ranged from 740 mm^2 to 2370 mm^2 across enrolled HCM patients. Typical myocardial T1 and T2 values were approximately 997 ms and 53 ms (median values across HCM patients), respectively.

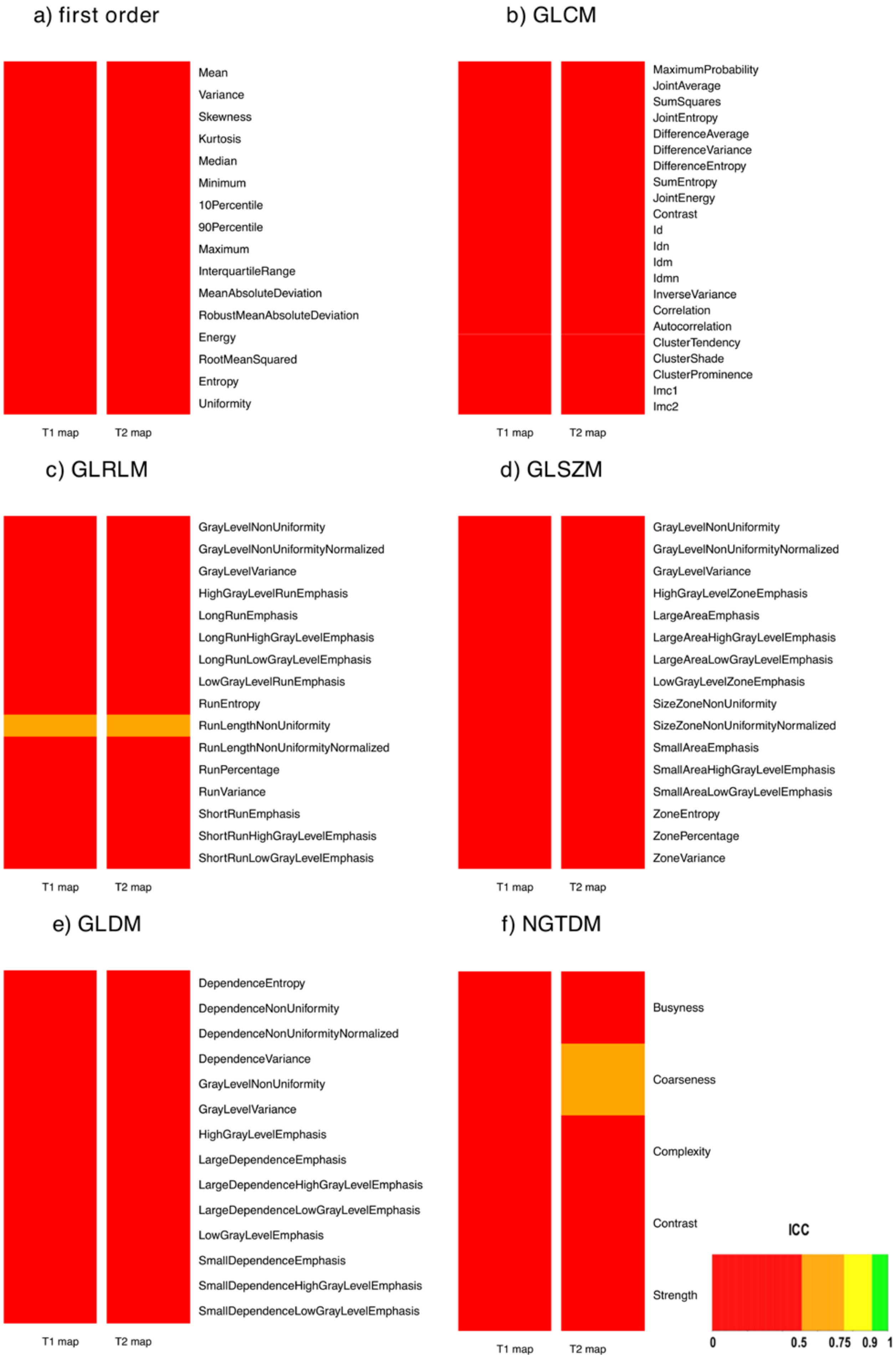

a) first order
Mean
Variance
Skewness
Kurtosis
Median
Minimum
10Percentile
90Percentile
Maximum
InterquartileRange
MeanAbsoluteDeviation
RobustMeanAbsoluteDeviation
Energy
RootMeanSquared
Entropy
Uniformity
T1 map
T2 map
b) GLCM
MaximumProbability
JointAverage
SumSquares
JointEntropy
DifferenceAverage
DifferenceVariance
DifferenceEntropy
SumEntropy
JointEnergy
Contrast
Id
Idn
Idm
Idmn
InverseVariance
Correlation
Autocorrelation
ClusterTendency
ClusterShade
ClusterProminence
Imc1
Imc2
T1 map
T2 map
c) GLRLM
GrayLevelNonUniformity
GrayLevelNonUniformityNormalized
GrayLevelVariance
HighGrayLevelRunEmphasis
LongRunEmphasis
LongRunHighGrayLevelEmphasis
LongRunLowGrayLevelEmphasis
LowGrayLevelRunEmphasis
RunEntropy
RunLengthNonUniformity
RunLengthNonUniformityNormalized
RunPercentage
RunVariance
ShortRunEmphasis
ShortRunHighGrayLevelEmphasis
ShortRunLowGrayLevelEmphasis
T1 map
T2 map
d) GLSZM
GrayLevelNonUniformity
GrayLevelNonUniformityNormalized
GrayLevelVariance
HighGrayLevelZoneEmphasis
LargeAreaEmphasis
LargeAreaHighGrayLevelEmphasis
LargeAreaLowGrayLevelEmphasis
LowGrayLevelZoneEmphasis
SizeZoneNonUniformity
SizeZoneNonUniformityNormalized
SmallAreaEmphasis
SmallAreaHighGrayLevelEmphasis
SmallAreaLowGrayLevelEmphasis
ZoneEntropy
ZonePercentage
ZoneVariance
T1 map
T2 map
e) GLDM
DependenceEntropy
DependenceNonUniformity
DependenceNonUniformityNormalized
DependenceVariance
GrayLevelNonUniformity
GrayLevelVariance
HighGrayLevelEmphasis
LargeDependenceEmphasis
LargeDependenceHighGrayLevelEmphasis
LargeDependenceLowGrayLevelEmphasis
LowGrayLevelEmphasis
SmallDependenceEmphasis
SmallDependenceHighGrayLevelEmphasis
SmallDependenceLowGrayLevelEmphasis
T1 map
T2 map
f) NGTDM
Busyness
Coarseness
Complexity
Contrast
Strength
T1 map
T2 map
ICC
0
0.5
0.75
0.9
1

Supplementary Figure S2. ICC results for effect C for both T1 and T2 maps. The heatmap of each radiomic features class (i.e., first order, GLCM, GLRLM, GLSZM, GLDM, and NGTDM) shows the degree of relative variability in radiomic features estimate when using different spatial filters with fixed bin width (i.e., 6 ms and 0.56 ms for T1 and T2 maps, respectively) and resampling voxel size (i.e., 2.1 mm for both T1 and T2 maps).

Supplementary Table S1. Median CV (%) of radiomic features estimate for effect C for both T1 and T2 maps. The median CV was calculated across subjects.

first order	T1	T2
Mean	123.38	137.01
Variance	157.96	162.00
Skewness	290.63	124.54
Kurtosis	102.27	83.10
Median	123.39	137.89
Minimum	179.51	323.76
10Percentile	129.29	148.96
90Percentile	119.12	126.82
Maximum	98.00	94.83
InterquartileRange	87.09	81.68
MeanAbsoluteDeviation	78.07	86.65
RobustMeanAbsoluteDeviation	88.39	84.91
Energy	147.94	161.71
RootMeanSquared	115.92	121.92
Entropy	22.16	22.65
Uniformity	68.06	69.71

GLCM	T1	T2
MaximumProbability	88.76	89.84
JointAverage	81.60	79.79
SumSquares	157.56	156.98
JointEntropy	15.78	16.98
DifferenceAverage	60.44	64.92
DifferenceVariance	127.76	132.98
DifferenceEntropy	20.72	23.57
SumEntropy	19.58	19.92
JointEnergy	103.58	101.07
Contrast	123.98	126.37
Id	27.61	36.75
Idn	2.01	2.04
Idm	38.71	52.74
Idmn	0.45	0.47
InverseVariance	32.53	46.06
Correlation	70.13	69.79
Autocorrelation	153.87	122.41
ClusterTendency	164.02	171.18
ClusterShade	240.89	258.39
ClusterProminence	235.08	239.88
Imc1	38.00	42.61
Imc2	10.38	12.14

NGTDM	T1	T2
Busyness	213.50	167.31
Coarseness	25.32	16.66
Complexity	170.55	155.95
Contrast	105.02	122.49
Strength	138.12	138.02

GLRLM	T1	T2
GrayLevelNonUniformity	56.94	59.79
GrayLevelNonUniformityNormalized	64.65	67.31
GrayLevelVariance	156.84	160.07
HighGrayLevelRunEmphasis	153.34	124.01
LongRunEmphasis	19.75	21.85
LongRunHighGrayLevelEmphasis	143.84	122.48
LongRunLowGrayLevelEmphasis	238.91	196.44
LowGrayLevelRunEmphasis	214.81	179.96
RunEntropy	15.69	15.61
RunLengthNonUniformity	15.03	16.41
RunLengthNonUniformityNormalized	9.62	10.82
RunPercentage	5.93	6.46
RunVariance	74.19	92.94
ShortRunEmphasis	4.27	4.76
ShortRunHighGrayLevelEmphasis	155.70	123.67
ShortRunLowGrayLevelEmphasis	207.87	175.45

GLSZM	T1	T2
GrayLevelNonUniformity	32.44	34.44
GrayLevelNonUniformityNormalized	56.05	60.46
GrayLevelVariance	154.66	154.80
HighGrayLevelZoneEmphasis	153.12	122.66
LargeAreaEmphasis	98.70	105.73
LargeAreaHighGrayLevelEmphasis	143.67	120.75
LargeAreaLowGrayLevelEmphasis	258.03	215.60
LowGrayLevelZoneEmphasis	202.41	170.09
SizeZoneNonUniformity	36.45	39.43
SizeZoneNonUniformityNormalized	19.03	22.57
SmallAreaEmphasis	9.36	11.92
SmallAreaHighGrayLevelEmphasis	159.11	121.66
SmallAreaLowGrayLevelEmphasis	173.29	152.38
ZoneEntropy	9.40	9.15
ZonePercentage	19.89	22.20
ZoneVariance	141.93	140.58

GLDM	T1	T2
DependenceEntropy	10.03	9.67
DependenceNonUniformity	31.40	33.09
DependenceNonUniformityNormalized	31.40	33.09
DependenceVariance	62.51	72.02
GrayLevelNonUniformity	68.06	69.71
GrayLevelVariance	157.95	161.99
HighGrayLevelEmphasis	153.44	124.29
LargeDependenceEmphasis	52.59	66.06
LargeDependenceHighGrayLevelEmphasis	131.67	121.00
LargeDependenceLowGrayLevelEmphasis	254.19	213.45
LowGrayLevelEmphasis	218.28	185.60
SmallDependenceEmphasis	22.65	24.83
SmallDependenceHighGrayLevelEmphasis	170.28	122.94
SmallDependenceLowGrayLevelEmphasis	156.56	144.56

Supplementary Table S2. Coefficient of linear correlation (r) between radiomic features estimates and voxel size for different bin widths (i.e., 3.60, 3.95, 4.30, 4.65, 5.00, 5.35, 5.70, 6.05, and 6.40 ms) for T1 maps. Significant ($p < 0.05$, adjusted for multiple comparisons using Bonferroni correction) correlations are highlighted in bold.

**shape**	**3.60**	**3.95**	**4.30**	**4.65**	**5.00**	**5.35**	**5.70**	**6.05**	**6.40**
Elongation	-0.13	-0.11	-0.11	-0.11	-0.11	-0.11	-0.11	-0.11	-0.11
MajorAxisLength	0.06	0.01	0.01	0.01	0.01	0.01	0.01	0.01	0.01
MaximumDiameter	0.06	0.02	0.02	0.02	0.02	0.02	0.02	0.02	0.02
MeshSurface	0.04	0.02	0.02	0.02	0.02	0.02	0.02	0.02	0.02
MinorAxisLength	-0.11	-0.14	-0.14	-0.14	-0.14	-0.14	-0.14	-0.14	-0.14
Perimeter	**-0.54**	**-0.61**	**-0.61**	**-0.61**	**-0.61**	**-0.61**	**-0.61**	**-0.61**	**-0.61**
PerimeterSurfaceRatio	-0.19	-0.25	-0.25	-0.25	-0.25	-0.25	-0.25	-0.25	-0.25
PixelSurface	0.07	0.06	0.06	0.06	0.06	0.06	0.06	0.06	0.06
Sphericity	**0.41**	**0.48**	**0.48**	**0.48**	**0.48**	**0.48**	**0.48**	**0.48**	**0.48**

**first order**	**3.60**	**3.95**	**4.30**	**4.65**	**5.00**	**5.35**	**5.70**	**6.05**	**6.40**
Mean	-0.01	0.00	0.00	0.00	0.00	0.00	0.00	0.00	0.00
Variance	0.12	0.16	0.16	0.16	0.16	0.16	0.16	0.16	0.16
Skewness	0.06	0.11	0.11	0.11	0.11	0.11	0.11	0.11	0.11
Kurtosis	0.13	0.14	0.14	0.14	0.14	0.14	0.14	0.14	0.14
Median	-0.01	0.00	0.00	0.00	0.00	0.00	0.00	0.00	0.00
Minimum	0.07	0.13	0.13	0.13	0.13	0.13	0.13	0.13	0.13
10Percentile	0.02	0.01	0.01	0.01	0.01	0.01	0.01	0.01	0.01
90Percentile	0.04	0.08	0.08	0.08	0.08	0.08	0.08	0.08	0.08
Maximum	0.01	0.06	0.06	0.06	0.06	0.06	0.06	0.06	0.06
InterquartileRange	0.01	0.03	0.03	0.03	0.03	0.03	0.03	0.03	0.03
MeanAbsoluteDeviation	0.09	0.12	0.12	0.12	0.12	0.12	0.12	0.12	0.12
RobustMeanAbsoluteDeviation	0.09	0.10	0.10	0.10	0.10	0.10	0.10	0.10	0.10
Energy	**-0.94**	**-0.93**	**-0.93**	**-0.93**	**-0.93**	**-0.93**	**-0.93**	**-0.93**	**-0.93**
RootMeanSquared	0.00	0.01	0.01	0.01	0.01	0.01	0.01	0.01	0.01
Entropy	**-0.55**	**-0.56**	**-0.49**	**-0.48**	**-0.51**	**-0.42**	**-0.39**	**-0.39**	-0.32
Uniformity	**0.48**	**0.49**	**0.41**	0.38	**0.43**	0.33	0.34	0.35	0.29

**GLCM**	**3.60**	**3.95**	**4.30**	**4.65**	**5.00**	**5.35**	**5.70**	**6.05**	**6.40**
MaximumProbability	**0.42**	**0.38**	0.21	**0.45**	0.30	0.27	0.26	0.25	0.26
JointAverage	-0.08	-0.12	-0.13	-0.13	-0.12	-0.12	-0.13	-0.13	-0.12
SumSquares	0.09	0.13	0.13	0.13	0.13	0.13	0.13	0.14	0.14
JointEntropy	**-0.93**	**-0.92**	**-0.91**	**-0.89**	**-0.88**	**-0.85**	**-0.83**	**-0.83**	**-0.78**
DifferenceAverage	**0.74**	**0.72**	**0.72**	**0.72**	**0.72**	**0.72**	**0.72**	**0.72**	**0.73**
DifferenceVariance	**0.44**	**0.43**	**0.43**	**0.43**	**0.43**	**0.43**	**0.43**	**0.43**	**0.43**
DifferenceEntropy	**0.72**	**0.69**	**0.70**	**0.71**	**0.72**	**0.72**	**0.75**	**0.76**	**0.76**
SumEntropy	**-0.83**	**-0.81**	**-0.80**	**-0.80**	**-0.79**	**-0.77**	**-0.77**	**-0.76**	**-0.75**
JointEnergy	**0.80**	**0.80**	**0.77**	**0.76**	**0.74**	**0.69**	**0.68**	**0.67**	**0.61**
Contrast	**0.50**	**0.49**	**0.49**	**0.49**	**0.49**	**0.49**	**0.49**	**0.49**	**0.49**
Id	**-0.85**	**-0.86**	**-0.84**	**-0.86**	**-0.86**	**-0.88**	**-0.86**	**-0.87**	**-0.88**
Idn	**-0.66**	**-0.68**	**-0.67**	**-0.68**	**-0.68**	**-0.68**	**-0.68**	**-0.67**	**-0.68**
Idm	**-0.81**	**-0.83**	**-0.81**	**-0.84**	**-0.83**	**-0.86**	**-0.84**	**-0.86**	**-0.87**
Idmn	**-0.63**	**-0.64**	**-0.64**	**-0.64**	**-0.64**	**-0.64**	**-0.64**	**-0.64**	**-0.64**
InverseVariance	**-0.74**	**-0.70**	**-0.72**	**-0.76**	**-0.74**	**-0.72**	**-0.74**	**-0.73**	**-0.75**
Correlation	**-0.80**	**-0.77**	**-0.77**	**-0.77**	**-0.77**	**-0.77**	**-0.77**	**-0.77**	**-0.77**
Autocorrelation	-0.08	-0.10	-0.11	-0.11	-0.10	-0.11	-0.11	-0.12	-0.10
ClusterTendency	-0.06	-0.01	-0.01	-0.01	-0.01	-0.01	-0.01	-0.01	-0.01
ClusterShade	0.08	0.11	0.11	0.11	0.11	0.11	0.11	0.11	0.10
ClusterProminence	0.06	0.10	0.10	0.10	0.10	0.10	0.10	0.10	0.10
Imc1	**-0.92**	**-0.92**	**-0.89**	**-0.88**	**-0.87**	**-0.85**	**-0.84**	**-0.83**	**-0.77**
Imc2	**0.71**	**0.70**	**0.71**	**0.70**	**0.68**	**0.68**	**0.67**	**0.69**	**0.61**

**NGTDM**	**3.60**	**3.95**	**4.30**	**4.65**	**5.00**	**5.35**	**5.70**	**6.05**	**6.40**
Busyness	-0.05	-0.01	-0.04	-0.04	-0.04	-0.14	-0.11	-0.12	-0.18
Coarseness	**0.73**	**0.72**	**0.76**	**0.74**	**0.73**	**0.77**	**0.78**	**0.75**	**0.79**
Complexity	0.23	0.26	0.26	0.24	0.27	0.27	0.29	0.30	0.30
Contrast	**0.50**	**0.50**	**0.50**	**0.53**	**0.53**	**0.56**	**0.55**	**0.57**	**0.58**
Strength	0.12	0.15	0.17	0.15	0.16	0.20	0.22	0.24	0.24

**GLRLM**	**3.60**	**3.95**	**4.30**	**4.65**	**5.00**	**5.35**	**5.70**	**6.05**	**6.40**
GrayLevelNonUniformity	**-0.90**	**-0.89**	**-0.89**	**-0.89**	**-0.89**	**-0.89**	**-0.89**	**-0.89**	**-0.89**
GrayLevelNonUniformityNormalized	**0.51**	**0.53**	**0.45**	**0.40**	**0.48**	0.36	**0.39**	**0.38**	0.30
GrayLevelVariance	0.12	0.15	0.15	0.15	0.15	0.15	0.15	0.15	0.15
HighGrayLevelRunEmphasis	-0.07	-0.09	-0.10	-0.10	-0.09	-0.10	-0.10	-0.10	-0.10
LongRunEmphasis	**-0.63**	**-0.71**	**-0.67**	**-0.72**	**-0.72**	**-0.72**	**-0.73**	**-0.75**	**-0.78**
LongRunHighGrayLevelEmphasis	-0.13	-0.16	-0.17	-0.17	-0.18	-0.19	-0.20	-0.21	-0.20
LongRunLowGrayLevelEmphasis	**0.40**	**0.44**	**0.46**	**0.43**	**0.40**	0.36	0.36	0.33	0.31
LowGrayLevelRunEmphasis	**0.44**	**0.49**	**0.54**	**0.50**	**0.48**	**0.45**	**0.45**	**0.43**	**0.42**
RunEntropy	**-0.75**	**-0.79**	**-0.72**	**-0.76**	**-0.79**	**-0.72**	**-0.73**	**-0.74**	**-0.73**
RunLengthNonUniformity	**-0.95**	**-0.94**	**-0.94**	**-0.94**	**-0.94**	**-0.94**	**-0.94**	**-0.94**	**-0.94**
RunLengthNonUniformityNormalized	**0.60**	**0.66**	**0.63**	**0.67**	**0.65**	**0.70**	**0.65**	**0.69**	**0.73**
RunPercentage	**0.62**	**0.70**	**0.67**	**0.71**	**0.70**	**0.73**	**0.71**	**0.73**	**0.78**
RunVariance	**-0.61**	**-0.69**	**-0.66**	**-0.71**	**-0.71**	**-0.69**	**-0.74**	**-0.74**	**-0.76**
ShortRunEmphasis	**0.60**	**0.67**	**0.63**	**0.67**	**0.65**	**0.70**	**0.66**	**0.69**	**0.74**
ShortRunHighGrayLevelEmphasis	-0.06	-0.08	-0.08	-0.09	-0.07	-0.07	-0.08	-0.08	-0.07
ShortRunLowGrayLevelEmphasis	**0.45**	**0.51**	**0.56**	**0.51**	**0.49**	**0.47**	**0.48**	**0.45**	**0.45**

**GLSZM**	**3.60**	**3.95**	**4.30**	**4.65**	**5.00**	**5.35**	**5.70**	**6.05**	**6.40**
GrayLevelNonUniformity	**-0.90**	**-0.89**	**-0.88**	**-0.89**	**-0.89**	**-0.89**	**-0.89**	**-0.89**	**-0.87**
GrayLevelNonUniformityNormalized	**0.54**	**0.58**	**0.49**	**0.39**	**0.47**	0.34	**0.38**	0.34	0.28
GrayLevelVariance	0.12	0.12	0.11	0.13	0.11	0.12	0.13	0.14	0.13
HighGrayLevelZoneEmphasis	-0.07	-0.09	-0.09	-0.10	-0.08	-0.09	-0.09	-0.09	-0.10
LargeAreaEmphasis	**-0.61**	**-0.72**	**-0.68**	**-0.70**	**-0.71**	**-0.70**	**-0.71**	**-0.74**	**-0.72**
LargeAreaHighGrayLevelEmphasis	-0.26	-0.36	-0.36	-0.36	**-0.39**	**-0.41**	**-0.43**	**-0.44**	**-0.45**
LargeAreaLowGrayLevelEmphasis	0.28	0.31	0.31	0.25	0.21	0.14	0.19	0.13	0.05
LowGrayLevelZoneEmphasis	**0.43**	**0.49**	**0.55**	**0.51**	**0.50**	**0.49**	**0.49**	**0.47**	**0.45**
SizeZoneNonUniformity	**-0.90**	**-0.87**	**-0.88**	**-0.88**	**-0.88**	**-0.87**	**-0.86**	**-0.83**	**-0.81**
SizeZoneNonUniformityNormalized	**0.56**	**0.62**	**0.57**	**0.61**	**0.60**	**0.67**	**0.56**	**0.64**	**0.64**
SmallAreaEmphasis	**0.57**	**0.63**	**0.58**	**0.63**	**0.61**	**0.68**	**0.57**	**0.65**	**0.65**
SmallAreaHighGrayLevelEmphasis	-0.03	-0.04	-0.04	-0.05	-0.02	-0.02	-0.03	-0.01	-0.03
SmallAreaLowGrayLevelEmphasis	**0.40**	**0.47**	**0.54**	**0.51**	**0.50**	**0.55**	**0.51**	**0.52**	**0.50**
ZoneEntropy	**-0.82**	**-0.85**	**-0.83**	**-0.84**	**-0.83**	**-0.83**	**-0.81**	**-0.83**	**-0.80**
ZonePercentage	**0.63**	**0.71**	**0.68**	**0.72**	**0.71**	**0.76**	**0.72**	**0.75**	**0.78**
ZoneVariance	**-0.55**	**-0.69**	**-0.63**	**-0.64**	**-0.67**	**-0.62**	**-0.66**	**-0.68**	**-0.64**

**GLDM**	**3.60**	**3.95**	**4.30**	**4.65**	**5.00**	**5.35**	**5.70**	**6.05**	**6.40**
DependenceEntropy	**-0.86**	**-0.89**	**-0.87**	**-0.87**	**-0.87**	**-0.86**	**-0.87**	**-0.85**	**-0.85**
DependenceNonUniformity	**-0.90**	**-0.88**	**-0.89**	**-0.89**	**-0.90**	**-0.89**	**-0.90**	**-0.89**	**-0.89**
DependenceNonUniformityNormalized	**0.61**	**0.66**	**0.65**	**0.67**	**0.67**	**0.72**	**0.67**	**0.67**	**0.72**
DependenceVariance	**-0.50**	**-0.59**	**-0.52**	**-0.52**	**-0.61**	**-0.52**	**-0.56**	**-0.53**	**-0.62**
GrayLevelNonUniformity	**-0.90**	**-0.88**	**-0.89**	**-0.89**	**-0.89**	**-0.89**	**-0.89**	**-0.89**	**-0.89**
GrayLevelVariance	0.12	0.16	0.16	0.16	0.16	0.16	0.16	0.16	0.16
HighGrayLevelEmphasis	-0.07	-0.09	-0.10	-0.10	-0.09	-0.10	-0.10	-0.11	-0.10
LargeDependenceEmphasis	**-0.60**	**-0.69**	**-0.64**	**-0.67**	**-0.69**	**-0.68**	**-0.68**	**-0.70**	**-0.76**
LargeDependenceHighGrayLevelEmphasis	-0.28	-0.37	-0.37	-0.34	**-0.38**	**-0.40**	**-0.42**	**-0.41**	**-0.40**
LargeDependenceLowGrayLevelEmphasis	0.23	0.26	0.24	0.20	0.16	0.11	0.19	0.15	0.08
LowGrayLevelEmphasis	**0.44**	**0.49**	**0.52**	**0.49**	**0.47**	**0.43**	**0.44**	**0.42**	**0.41**
SmallDependenceEmphasis	**0.62**	**0.68**	**0.65**	**0.70**	**0.68**	**0.74**	**0.67**	**0.73**	**0.75**
SmallDependenceHighGrayLevelEmphasis	0.02	0.02	0.02	0.01	0.05	0.05	0.04	0.06	0.05
SmallDependenceLowGrayLevelEmphasis	**0.46**	**0.54**	**0.60**	**0.57**	**0.55**	**0.59**	**0.58**	**0.60**	**0.59**

Supplementary Table S3. Coefficient of linear correlation (r) between radiomic features estimates and bin width for different voxel sizes (i.e., 1.8, 1.9, 2.0, 2.1, 2.2, 2.3, and 2.4 mm) for T1 maps. Significant ($p < 0.05$, adjusted for multiple comparisons using Bonferroni correction) correlations are highlighted in bold.

**Shape**	**1.80**	**1.90**	**2.00**	**2.10**	**2.20**	**2.30**	**2.40**
Elongation	0.10	0.08	-0.06	0.11	-0.10	0.09	-0.01
MajorAxisLength	**0.14**	0.00	-0.02	0.05	-0.08	0.13	0.12
MaximumDiameter	0.09	-0.10	-0.08	-0.06	0.12	0.12	-0.01
MeshSurface	-0.08	0.05	0.00	-0.03	0.03	0.02	0.03
MinorAxisLength	0.12	0.13	0.08	-0.07	0.12	0.02	-0.04
Perimeter	0.02	-0.03	0.12	0.12	0.12	0.05	-0.05
PerimeterSurfaceRatio	0.08	0.00	0.00	0.09	0.00	0.10	-0.03
PixelSurface	0.01	0.05	0.01	-0.01	0.07	-0.06	-0.08
Sphericity	-0.02	0.07	-0.13	-0.06	-0.03	0.03	0.00

**first order**	**1.80**	**1.90**	**2.00**	**2.10**	**2.20**	**2.30**	**2.40**
Mean	0.11	-0.09	0.09	0.09	0.11	0.11	0.03
Variance	0.03	0.02	0.04	0.02	-0.02	-0.05	-0.01
Skewness	0.02	-0.04	-0.04	-0.01	0.00	-0.01	0.01
Kurtosis	0.11	0.11	0.03	0.02	0.00	0.12	0.04
Median	0.11	-0.10	0.11	-0.10	0.11	-0.10	-0.07
Minimum	-0.05	-0.11	-0.12	0.06	0.01	-0.10	-0.08
10Percentile	0.09	-0.08	-0.12	-0.11	-0.10	-0.11	0.08
90Percentile	-0.10	0.03	0.10	0.09	0.09	0.10	0.11
Maximum	0.11	-0.09	0.12	0.04	-0.09	0.11	0.13
InterquartileRange	0.01	-0.04	0.01	-0.07	-0.08	-0.06	-0.01
MeanAbsoluteDeviation	-0.03	-0.01	0.01	-0.04	-0.06	0.04	0.06
RobustMeanAbsoluteDeviation	0.01	0.03	-0.02	-0.01	-0.01	0.03	-0.04
Energy	0.02	-0.06	-0.07	-0.04	-0.05	0.00	-0.08
RootMeanSquared	-0.07	-0.10	0.09	0.11	0.08	0.02	0.10
Entropy	**-1.00**	**-0.99**	**-0.99**	**-0.99**	**-0.99**	**-0.99**	**-0.99**
Uniformity	**0.97**	**0.97**	**0.97**	**0.97**	**0.97**	**0.97**	**0.97**

**GLCM**	**1.80**	**1.90**	**2.00**	**2.10**	**2.20**	**2.30**	**2.40**
MaximumProbability	**0.84**	**0.82**	**0.80**	**0.74**	**0.83**	**0.78**	**0.77**
JointAverage	**-0.93**	**-0.93**	**-0.93**	**-0.93**	**-0.93**	**-0.92**	**-0.93**
SumSquares	**-0.75**	**-0.77**	**-0.79**	**-0.73**	**-0.76**	**-0.73**	**-0.73**
JointEntropy	**-0.95**	**-0.95**	**-0.94**	**-0.93**	**-0.93**	**-0.93**	**-0.93**
DifferenceAverage	**-0.93**	**-0.92**	**-0.93**	**-0.92**	**-0.93**	**-0.92**	**-0.91**
DifferenceVariance	**-0.74**	**-0.70**	**-0.74**	**-0.69**	**-0.74**	**-0.70**	**-0.67**
DifferenceEntropy	**-1.00**	**-1.00**	**-1.00**	**-0.99**	**-0.99**	**-0.99**	**-0.99**
SumEntropy	**-0.99**	**-0.99**	**-0.99**	**-0.98**	**-0.98**	**-0.98**	**-0.98**
JointEnergy	**0.94**	**0.94**	**0.93**	**0.92**	**0.93**	**0.93**	**0.93**
Contrast	**-0.72**	**-0.69**	**-0.73**	**-0.67**	**-0.72**	**-0.68**	**-0.66**
Id	**0.99**	**0.99**	**0.99**	**0.99**	**0.99**	**0.99**	**0.99**
Idn	**0.31**	**0.38**	**0.39**	**0.43**	**0.34**	**0.35**	**0.44**
Idm	**0.98**	**0.98**	**0.98**	**0.98**	**0.98**	**0.98**	**0.98**
Idmn	0.16	**0.32**	**0.28**	**0.34**	0.25	0.27	**0.36**
InverseVariance	**0.97**	**0.97**	**0.97**	**0.97**	**0.97**	**0.95**	**0.95**
Correlation	**-0.34**	-0.18	-0.17	-0.19	-0.12	-0.15	-0.23
Autocorrelation	**-0.78**	**-0.79**	**-0.80**	**-0.80**	**-0.79**	**-0.76**	**-0.81**
ClusterTendency	**-0.75**	**-0.78**	**-0.79**	**-0.74**	**-0.76**	**-0.74**	**-0.74**
ClusterShade	**0.33**	0.27	**0.32**	**0.30**	0.25	**0.31**	0.18
ClusterProminence	**-0.46**	**-0.52**	**-0.53**	**-0.47**	**-0.50**	**-0.47**	**-0.44**
Imc1	**0.95**	**0.96**	**0.96**	**0.97**	**0.97**	**0.97**	**0.98**
Imc2	**-0.87**	**-0.86**	**-0.85**	**-0.84**	**-0.82**	**-0.82**	**-0.81**

**NGTDM**	**1.80**	**1.90**	**2.00**	**2.10**	**2.20**	**2.30**	**2.40**
Busyness	**0.84**	**0.82**	**0.81**	**0.87**	**0.82**	**0.85**	**0.85**
Coarseness	**0.65**	**0.68**	**0.69**	**0.66**	**0.70**	**0.74**	**0.69**
Complexity	**-0.69**	**-0.68**	**-0.72**	**-0.65**	**-0.68**	**-0.68**	**-0.67**
Contrast	**-0.74**	**-0.72**	**-0.69**	**-0.61**	**-0.66**	**-0.58**	**-0.57**
Strength	**-0.80**	**-0.84**	**-0.84**	**-0.84**	**-0.84**	**-0.82**	**-0.83**

**GLRLM**	**1.80**	**1.90**	**2.00**	**2.10**	**2.20**	**2.30**	**2.40**
GrayLevelNonUniformity	**0.93**	**0.93**	**0.92**	**0.92**	**0.92**	**0.93**	**0.92**
GrayLevelNonUniformityNormalized	**0.97**	**0.97**	**0.97**	**0.97**	**0.97**	**0.97**	**0.97**
GrayLevelVariance	**-0.75**	**-0.77**	**-0.78**	**-0.73**	**-0.75**	**-0.72**	**-0.72**
HighGrayLevelRunEmphasis	**-0.78**	**-0.80**	**-0.80**	**-0.80**	**-0.80**	**-0.76**	**-0.81**
LongRunEmphasis	**0.91**	**0.90**	**0.91**	**0.89**	**0.89**	**0.88**	**0.88**
LongRunHighGrayLevelEmphasis	**-0.79**	**-0.80**	**-0.81**	**-0.80**	**-0.80**	**-0.77**	**-0.81**
LongRunLowGrayLevelEmphasis	**0.66**	**0.71**	**0.66**	**0.65**	**0.65**	**0.63**	**0.49**
LowGrayLevelRunEmphasis	**0.72**	**0.72**	**0.68**	**0.70**	**0.73**	**0.68**	**0.65**
RunEntropy	**-0.99**	**-0.99**	**-0.99**	**-0.99**	**-0.99**	**-0.99**	**-0.99**
RunLengthNonUniformity	**-0.89**	**-0.88**	**-0.88**	**-0.85**	**-0.85**	**-0.85**	**-0.84**
RunLengthNonUniformityNormalized	**-0.94**	**-0.92**	**-0.92**	**-0.90**	**-0.90**	**-0.90**	**-0.89**
RunPercentage	**-0.93**	**-0.92**	**-0.93**	**-0.90**	**-0.91**	**-0.89**	**-0.89**
RunVariance	**0.89**	**0.88**	**0.88**	**0.87**	**0.87**	**0.85**	**0.85**
ShortRunEmphasis	**-0.93**	**-0.92**	**-0.92**	**-0.90**	**-0.90**	**-0.89**	**-0.89**
ShortRunHighGrayLevelEmphasis	**-0.78**	**-0.80**	**-0.80**	**-0.80**	**-0.79**	**-0.76**	**-0.81**
ShortRunLowGrayLevelEmphasis	**0.73**	**0.73**	**0.69**	**0.70**	**0.75**	**0.68**	**0.69**

**GLSZM**	**1.80**	**1.90**	**2.00**	**2.10**	**2.20**	**2.30**	**2.40**
GrayLevelNonUniformity	**0.93**	**0.93**	**0.92**	**0.92**	**0.92**	**0.93**	**0.92**
GrayLevelNonUniformityNormalized	**0.96**	**0.97**	**0.96**	**0.96**	**0.96**	**0.96**	**0.96**
GrayLevelVariance	**-0.75**	**-0.77**	**-0.78**	**-0.73**	**-0.76**	**-0.72**	**-0.72**
HighGrayLevelZoneEmphasis	**-0.79**	**-0.80**	**-0.80**	**-0.80**	**-0.80**	**-0.77**	**-0.82**
LargeAreaEmphasis	**0.86**	**0.85**	**0.85**	**0.86**	**0.84**	**0.84**	**0.82**
LargeAreaHighGrayLevelEmphasis	**-0.78**	**-0.77**	**-0.79**	**-0.80**	**-0.79**	**-0.77**	**-0.80**
LargeAreaLowGrayLevelEmphasis	**0.61**	**0.68**	**0.65**	**0.62**	**0.62**	**0.58**	**0.43**
LowGrayLevelZoneEmphasis	**0.78**	**0.76**	**0.72**	**0.74**	**0.79**	**0.73**	**0.76**
SizeZoneNonUniformity	**-0.91**	**-0.88**	**-0.89**	**-0.86**	**-0.85**	**-0.84**	**-0.85**
SizeZoneNonUniformityNormalized	**-0.92**	**-0.89**	**-0.90**	**-0.87**	**-0.87**	**-0.86**	**-0.86**
SmallAreaEmphasis	**-0.91**	**-0.89**	**-0.90**	**-0.87**	**-0.87**	**-0.85**	**-0.85**
SmallAreaHighGrayLevelEmphasis	**-0.77**	**-0.80**	**-0.80**	**-0.79**	**-0.79**	**-0.76**	**-0.81**
SmallAreaLowGrayLevelEmphasis	**0.67**	**0.65**	**0.59**	**0.60**	**0.77**	**0.63**	**0.65**
ZoneEntropy	**-0.94**	**-0.93**	**-0.94**	**-0.93**	**-0.94**	**-0.94**	**-0.93**
ZonePercentage	**-0.94**	**-0.93**	**-0.93**	**-0.92**	**-0.91**	**-0.90**	**-0.90**
ZoneVariance	**0.82**	**0.79**	**0.78**	**0.80**	**0.77**	**0.79**	**0.73**

**GLDM**	**1.80**	**1.90**	**2.00**	**2.10**	**2.20**	**2.30**	**2.40**
DependenceEntropy	**-0.93**	**-0.91**	**-0.93**	**-0.91**	**-0.92**	**-0.91**	**-0.91**
DependenceNonUniformity	**-0.93**	**-0.91**	**-0.91**	**-0.89**	**-0.89**	**-0.88**	**-0.88**
DependenceNonUniformityNormalized	**-0.94**	**-0.93**	**-0.94**	**-0.92**	**-0.92**	**-0.91**	**-0.90**
DependenceVariance	**0.87**	**0.82**	**0.83**	**0.78**	**0.82**	**0.79**	**0.80**
GrayLevelNonUniformity	**0.92**	**0.92**	**0.92**	**0.92**	**0.92**	**0.92**	**0.92**
GrayLevelVariance	**-0.75**	**-0.77**	**-0.78**	**-0.72**	**-0.75**	**-0.71**	**-0.71**
HighGrayLevelEmphasis	**-0.78**	**-0.80**	**-0.80**	**-0.80**	**-0.79**	**-0.76**	**-0.81**
LargeDependenceEmphasis	**0.91**	**0.90**	**0.90**	**0.87**	**0.88**	**0.87**	**0.86**
LargeDependenceHighGrayLevelEmphasis	**-0.81**	**-0.79**	**-0.79**	**-0.81**	**-0.80**	**-0.77**	**-0.79**
LargeDependenceLowGrayLevelEmphasis	**0.54**	**0.63**	**0.57**	**0.48**	**0.45**	**0.48**	0.23
LowGrayLevelEmphasis	**0.68**	**0.70**	**0.66**	**0.67**	**0.68**	**0.64**	**0.56**
SmallDependenceEmphasis	**-0.95**	**-0.92**	**-0.93**	**-0.91**	**-0.91**	**-0.90**	**-0.89**
SmallDependenceHighGrayLevelEmphasis	**-0.76**	**-0.80**	**-0.79**	**-0.78**	**-0.78**	**-0.75**	**-0.80**
SmallDependenceLowGrayLevelEmphasis	**0.62**	**0.58**	**0.50**	**0.53**	**0.72**	**0.58**	**0.57**

Supplementary Table S4. Coefficient of linear correlation (r) between radiomic features estimates and voxel size for different bin widths (i.e., 0.49, 0.50, 0.51, 0.52, 0.53, 0.54, 0.55, 0.56, and 0.57 ms) for T2 maps. Significant ($p < 0.05$, adjusted for multiple comparisons using Bonferroni correction) correlations are highlighted in bold.

**Shape**	**0.49**	**0.50**	**0.51**	**0.52**	**0.53**	**0.54**	**0.55**	**0.56**	**0.57**
Elongation	0.01	-0.03	-0.03	-0.03	-0.03	-0.03	-0.03	-0.03	-0.03
MajorAxisLength	-0.03	0.00	0.00	0.00	0.00	0.00	0.00	0.00	0.00
MaximumDiameter	0.13	0.07	0.07	0.07	0.07	0.07	0.07	0.07	0.07
MeshSurface	-0.10	-0.13	-0.13	-0.13	-0.13	-0.13	-0.13	-0.13	-0.13
MinorAxisLength	0.00	-0.04	-0.04	-0.04	-0.04	-0.04	-0.04	-0.04	-0.04
Perimeter	**-0.51**	**-0.62**	**-0.62**	**-0.62**	**-0.62**	**-0.62**	**-0.62**	**-0.62**	**-0.62**
PerimeterSurfaceRatio	-0.10	-0.11	-0.11	-0.11	-0.11	-0.11	-0.11	-0.11	-0.11
PixelSurface	-0.10	-0.12	-0.12	-0.12	-0.12	-0.12	-0.12	-0.12	-0.12
Sphericity	0.24	0.28	0.28	0.28	0.28	0.28	0.28	0.28	0.28

**first order**	**0.49**	**0.50**	**0.51**	**0.52**	**0.53**	**0.54**	**0.55**	**0.56**	**0.57**
Mean	0.00	0.05	0.05	0.05	0.05	0.05	0.05	0.05	0.05
Variance	0.06	0.12	0.12	0.12	0.12	0.12	0.12	0.12	0.12
Skewness	0.06	0.14	0.14	0.14	0.14	0.14	0.14	0.14	0.14
Kurtosis	0.06	0.11	0.11	0.11	0.11	0.11	0.11	0.11	0.11
Median	-0.12	-0.08	-0.08	-0.08	-0.08	-0.08	-0.08	-0.08	-0.08
Minimum	0.04	0.08	0.08	0.08	0.08	0.08	0.08	0.08	0.08
10Percentile	-0.07	-0.04	-0.04	-0.04	-0.04	-0.04	-0.04	-0.04	-0.04
90Percentile	0.06	0.14	0.14	0.14	0.14	0.14	0.14	0.14	0.14
Maximum	-0.05	0.03	0.03	0.03	0.03	0.03	0.03	0.03	0.03
InterquartileRange	-0.03	-0.11	-0.11	-0.11	-0.11	-0.11	-0.11	-0.11	-0.11
MeanAbsoluteDeviation	0.07	0.10	0.10	0.10	0.10	0.10	0.10	0.10	0.10
RobustMeanAbsoluteDeviation	0.07	0.05	0.05	0.05	0.05	0.05	0.05	0.05	0.05
Energy	**-0.94**	**-0.92**	**-0.92**	**-0.92**	**-0.92**	**-0.92**	**-0.92**	**-0.92**	**-0.92**
RootMeanSquared	0.01	0.07	0.07	0.07	0.07	0.07	0.07	0.07	0.07
Entropy	**-0.47**	**-0.43**	**-0.42**	**-0.41**	**-0.38**	**-0.42**	**-0.40**	**-0.41**	-0.34
Uniformity	**0.41**	**0.41**	0.37	**0.38**	0.36	**0.38**	0.37	**0.40**	0.33

**GLCM**	**0.49**	**0.50**	**0.51**	**0.52**	**0.53**	**0.54**	**0.55**	**0.56**	**0.57**
MaximumProbability	**0.50**	**0.52**	**0.46**	**0.48**	**0.47**	**0.43**	0.34	**0.47**	**0.38**
JointAverage	-0.01	-0.07	-0.08	-0.06	-0.08	-0.07	-0.07	-0.05	-0.08
SumSquares	0.06	0.13	0.13	0.13	0.13	0.13	0.13	0.13	0.14
JointEntropy	**-0.91**	**-0.92**	**-0.91**	**-0.91**	**-0.90**	**-0.89**	**-0.88**	**-0.89**	**-0.88**
DifferenceAverage	**0.83**	**0.82**	**0.82**	**0.82**	**0.83**	**0.82**	**0.83**	**0.83**	**0.83**
DifferenceVariance	**0.41**	**0.40**	**0.40**	**0.40**	**0.40**	**0.41**	**0.40**	**0.40**	**0.41**
DifferenceEntropy	**0.82**	**0.82**	**0.81**	**0.82**	**0.82**	**0.81**	**0.82**	**0.83**	**0.81**
SumEntropy	**-0.81**	**-0.83**	**-0.82**	**-0.81**	**-0.82**	**-0.82**	**-0.80**	**-0.81**	**-0.80**
JointEnergy	**0.81**	**0.81**	**0.79**	**0.81**	**0.78**	**0.75**	**0.75**	**0.78**	**0.74**
Contrast	**0.58**	**0.57**	**0.57**	**0.57**	**0.57**	**0.57**	**0.57**	**0.57**	**0.57**
Id	**-0.83**	**-0.82**	**-0.81**	**-0.81**	**-0.85**	**-0.84**	**-0.85**	**-0.83**	**-0.86**
Idn	**-0.56**	**-0.49**	**-0.51**	**-0.49**	**-0.50**	**-0.49**	**-0.50**	**-0.50**	**-0.51**
Idm	**-0.79**	**-0.77**	**-0.77**	**-0.77**	**-0.82**	**-0.81**	**-0.82**	**-0.80**	**-0.83**
Idmn	**-0.58**	**-0.53**	**-0.55**	**-0.52**	**-0.54**	**-0.53**	**-0.53**	**-0.53**	**-0.54**
InverseVariance	**-0.74**	**-0.74**	**-0.75**	**-0.76**	**-0.78**	**-0.77**	**-0.77**	**-0.82**	**-0.74**
Correlation	**-0.83**	**-0.82**	**-0.82**	**-0.83**	**-0.83**	**-0.83**	**-0.83**	**-0.83**	**-0.83**
Autocorrelation	-0.01	-0.06	-0.07	-0.05	-0.07	-0.07	-0.06	-0.04	-0.06
ClusterTendency	-0.15	-0.18	-0.18	-0.18	-0.18	-0.18	-0.18	-0.18	-0.17
ClusterShade	-0.04	0.06	0.06	0.06	0.06	0.06	0.06	0.06	0.06
ClusterProminence	-0.05	0.05	0.05	0.05	0.05	0.05	0.05	0.05	0.05
Imc1	**-0.90**	**-0.91**	**-0.90**	**-0.90**	**-0.88**	**-0.88**	**-0.88**	**-0.87**	**-0.87**
Imc2	**0.76**	**0.78**	**0.78**	**0.77**	**0.75**	**0.78**	**0.77**	**0.74**	**0.76**

**NGTDM**	**0.49**	**0.50**	**0.51**	**0.52**	**0.53**	**0.54**	**0.55**	**0.56**	**0.57**
Busyness	-0.16	-0.14	-0.12	-0.14	-0.14	-0.13	-0.15	-0.15	-0.15
Coarseness	**0.77**	**0.79**	**0.76**	**0.77**	**0.81**	**0.78**	**0.76**	**0.77**	**0.79**
Complexity	0.00	0.18	0.13	0.13	0.17	0.13	0.13	0.16	0.14
Contrast	**0.75**	**0.75**	**0.76**	**0.73**	**0.74**	**0.74**	**0.76**	**0.74**	**0.75**
Strength	0.07	0.22	0.18	0.21	0.21	0.19	0.19	0.20	0.19

**GLRLM**	**0.49**	**0.50**	**0.51**	**0.52**	**0.53**	**0.54**	**0.55**	**0.56**	**0.57**
GrayLevelNonUniformity	**-0.94**	**-0.94**	**-0.94**	**-0.94**	**-0.93**	**-0.93**	**-0.93**	**-0.94**	**-0.94**
GrayLevelNonUniformityNormalized	**0.42**	**0.43**	**0.39**	**0.41**	**0.38**	**0.40**	**0.40**	**0.42**	0.35
GrayLevelVariance	0.05	0.11	0.11	0.11	0.11	0.11	0.11	0.11	0.11
HighGrayLevelRunEmphasis	0.01	-0.04	-0.05	-0.03	-0.05	-0.04	-0.04	-0.02	-0.04
LongRunEmphasis	**-0.56**	**-0.56**	**-0.59**	**-0.58**	**-0.63**	**-0.66**	**-0.70**	**-0.61**	**-0.65**
LongRunHighGrayLevelEmphasis	-0.04	-0.10	-0.11	-0.10	-0.12	-0.12	-0.11	-0.08	-0.10
LongRunLowGrayLevelEmphasis	0.15	0.16	0.21	0.12	0.22	0.13	0.19	0.13	0.17
LowGrayLevelRunEmphasis	0.24	0.22	0.26	0.22	0.25	0.19	0.26	0.21	0.25
RunEntropy	**-0.72**	**-0.72**	**-0.73**	**-0.73**	**-0.69**	**-0.72**	**-0.73**	**-0.69**	**-0.68**
RunLengthNonUniformity	**-0.95**	**-0.94**	**-0.94**	**-0.94**	**-0.94**	**-0.95**	**-0.94**	**-0.94**	**-0.94**
RunLengthNonUniformityNormalized	**0.49**	**0.49**	**0.55**	**0.54**	**0.56**	**0.56**	**0.61**	**0.53**	**0.60**
RunPercentage	**0.54**	**0.54**	**0.58**	**0.57**	**0.61**	**0.63**	**0.67**	**0.59**	**0.64**
RunVariance	**-0.57**	**-0.56**	**-0.58**	**-0.57**	**-0.64**	**-0.68**	**-0.71**	**-0.61**	**-0.64**
ShortRunEmphasis	**0.50**	**0.50**	**0.56**	**0.54**	**0.56**	**0.57**	**0.61**	**0.54**	**0.60**
ShortRunHighGrayLevelEmphasis	0.02	-0.03	-0.03	-0.02	-0.03	-0.03	-0.02	-0.01	-0.03
ShortRunLowGrayLevelEmphasis	0.25	0.23	0.27	0.24	0.26	0.20	0.27	0.23	0.27

**GLSZM**	**0.49**	**0.50**	**0.51**	**0.52**	**0.53**	**0.54**	**0.55**	**0.56**	**0.57**
GrayLevelNonUniformity	**-0.93**	**-0.93**	**-0.93**	**-0.92**	**-0.92**	**-0.93**	**-0.93**	**-0.92**	**-0.93**
GrayLevelNonUniformityNormalized	**0.38**	**0.40**	**0.39**	**0.40**	**0.39**	**0.39**	0.36	**0.42**	0.37
GrayLevelVariance	0.02	0.09	0.09	0.09	0.08	0.09	0.08	0.08	0.07
HighGrayLevelZoneEmphasis	0.00	-0.05	-0.05	-0.04	-0.05	-0.05	-0.05	-0.03	-0.05
LargeAreaEmphasis	**-0.53**	**-0.52**	**-0.60**	**-0.54**	**-0.62**	**-0.62**	**-0.65**	**-0.60**	**-0.62**
LargeAreaHighGrayLevelEmphasis	-0.19	-0.23	-0.29	-0.29	-0.31	-0.31	-0.29	-0.24	-0.25
LargeAreaLowGrayLevelEmphasis	-0.01	0.07	0.08	-0.02	0.11	0.03	0.06	-0.02	0.05
LowGrayLevelZoneEmphasis	0.28	0.24	0.25	0.28	0.24	0.21	0.27	0.25	0.27
SizeZoneNonUniformity	**-0.90**	**-0.88**	**-0.89**	**-0.89**	**-0.88**	**-0.89**	**-0.88**	**-0.87**	**-0.88**
SizeZoneNonUniformityNormalized	**0.43**	**0.45**	**0.53**	**0.46**	**0.43**	**0.46**	**0.58**	0.36	**0.56**
SmallAreaEmphasis	**0.43**	**0.45**	**0.52**	**0.45**	**0.44**	**0.46**	**0.59**	0.37	**0.56**
SmallAreaHighGrayLevelEmphasis	0.04	0.00	0.01	0.02	0.00	0.00	0.00	0.02	0.00
SmallAreaLowGrayLevelEmphasis	**0.32**	0.25	0.27	0.37	0.24	0.25	0.29	0.29	0.27
ZoneEntropy	**-0.78**	**-0.79**	**-0.82**	**-0.78**	**-0.76**	**-0.78**	**-0.84**	**-0.78**	**-0.82**
ZonePercentage	**0.54**	**0.54**	**0.60**	**0.57**	**0.60**	**0.62**	**0.68**	**0.55**	**0.67**
ZoneVariance	**-0.48**	**-0.48**	**-0.56**	**-0.52**	**-0.59**	**-0.58**	**-0.60**	**-0.59**	**-0.55**

**GLDM**	**0.49**	**0.50**	**0.51**	**0.52**	**0.53**	**0.54**	**0.55**	**0.56**	**0.57**
DependenceEntropy	**-0.83**	**-0.86**	**-0.87**	**-0.85**	**-0.84**	**-0.84**	**-0.88**	**-0.85**	**-0.85**
DependenceNonUniformity	**-0.91**	**-0.90**	**-0.91**	**-0.91**	**-0.90**	**-0.91**	**-0.90**	**-0.90**	**-0.91**
DependenceNonUniformityNormalized	**0.53**	**0.53**	**0.58**	**0.58**	**0.56**	**0.60**	**0.65**	**0.54**	**0.64**
DependenceVariance	**-0.45**	**-0.46**	**-0.45**	**-0.50**	**-0.53**	**-0.54**	**-0.53**	**-0.59**	**-0.45**
GrayLevelNonUniformity	**-0.93**	**-0.93**	**-0.94**	**-0.94**	**-0.93**	**-0.93**	**-0.93**	**-0.93**	**-0.94**
GrayLevelVariance	0.07	0.12	0.12	0.12	0.12	0.12	0.12	0.12	0.13
HighGrayLevelEmphasis	0.01	-0.04	-0.05	-0.03	-0.05	-0.04	-0.04	-0.02	-0.04
LargeDependenceEmphasis	**-0.53**	**-0.53**	**-0.56**	**-0.56**	**-0.61**	**-0.62**	**-0.65**	**-0.61**	**-0.60**
LargeDependenceHighGrayLevelEmphasis	-0.19	-0.25	-0.26	-0.27	-0.30	-0.30	-0.28	-0.24	-0.24
LargeDependenceLowGrayLevelEmphasis	-0.01	0.07	0.05	-0.04	0.10	0.03	0.05	-0.05	0.06
LowGrayLevelEmphasis	0.22	0.21	0.25	0.20	0.25	0.18	0.25	0.20	0.25
SmallDependenceEmphasis	**0.51**	**0.51**	**0.58**	**0.54**	**0.54**	**0.57**	**0.65**	**0.49**	**0.64**
SmallDependenceHighGrayLevelEmphasis	0.09	0.06	0.07	0.08	0.07	0.07	0.07	0.07	0.07
SmallDependenceLowGrayLevelEmphasis	**0.34**	0.31	0.32	**0.40**	0.29	0.29	0.34	0.32	0.34

Supplementary Table S5. Coefficient of linear correlation (r) between radiomic features estimates and bin width for different voxel sizes (i.e., 1.8, 1.9, 2.0, 2.1, 2.2, 2.3, and 2.4 mm) for T2 maps. Significant ($p < 0.05$, adjusted for multiple comparisons using Bonferroni correction) correlations are highlighted in bold.

**shape**	**1,80**	**1,90**	**2,00**	**2,10**	**2,20**	**2,30**	**2,40**
Elongation	0.06	0.12	0.12	0.12	0.13	-0.09	0.12
MajorAxisLength	-0.03	-0.07	0.10	-0.07	0.08	-0.03	0.09
MaximumDiameter	-0.07	-0.08	-0.06	-0.06	-0.04	0.06	-0.07
MeshSurface	0.06	0.06	0.10	0.08	0.04	-0.02	0.02
MinorAxisLength	0.04	0.01	0.11	-0.07	0.12	0.09	0.12
Perimeter	-0.07	-0.08	0.07	0.04	0.05	0.03	-0.03
PerimeterSurfaceRatio	0.08	0.08	0.04	-0.05	0.09	-0.01	0.07
PixelSurface	0.00	0.09	0.09	0.06	0.05	-0.06	0.05
Sphericity	0.05	-0.09	0.09	0.09	0.04	0.10	-0.04

**first order**	**1.80**	**1.90**	**2.00**	**2.10**	**2.20**	**2.30**	**2.40**
Mean	-0.08	-0.11	-0.10	0.00	-0.10	0.08	0.10
Variance	0.00	0.06	0.04	0.02	0.04	0.08	-0.06
Skewness	0.08	-0.01	-0.06	-0.09	-0.02	0.04	0.03
Kurtosis	0.04	0.01	-0.06	-0.11	0.04	0.05	0.00
Median	-0.11	0.10	0.07	0.11	-0.07	-0.06	-0.10
Minimum	-0.14	-0.14	-0.14	-0.13	-0.12	0.08	-0.14
10Percentile	-0.11	0.10	0.09	-0.06	0.08	-0.11	0.09
90Percentile	-0.10	-0.11	-0.11	0.10	0.09	0.00	0.05
Maximum	0.13	0.10	-0.11	-0.11	0.09	0.12	0.00
InterquartileRange	0.04	0.08	0.00	0.10	-0.07	0.05	0.09
MeanAbsoluteDeviation	-0.03	0.00	0.02	0.06	0.03	0.09	-0.03
RobustMeanAbsoluteDeviation	-0.06	0.04	0.07	-0.09	0.07	0.09	0.06
Energy	0.07	-0.01	-0.07	-0.04	0.00	-0.04	-0.01
RootMeanSquared	-0.11	-0.01	0.10	-0.02	0.08	0.11	-0.10
Entropy	**-0.99**	**-0.99**	**-0.99**	**-0.98**	**-0.98**	**-0.98**	**-0.97**
Uniformity	**0.97**	**0.95**	**0.96**	**0.95**	**0.94**	**0.94**	**0.93**

**GLCM**	**1.80**	**1.90**	**2.00**	**2.10**	**2.20**	**2.30**	**2.40**
MaximumProbability	**0.62**	**0.39**	**0.52**	**0.58**	**0.28**	**0.39**	**0.31**
JointAverage	**-0.94**	**-0.94**	**-0.94**	**-0.94**	**-0.94**	**-0.93**	**-0.93**
SumSquares	**-0.79**	**-0.81**	**-0.77**	**-0.81**	**-0.81**	**-0.81**	**-0.78**
JointEntropy	**-0.97**	**-0.96**	**-0.95**	**-0.94**	**-0.92**	**-0.93**	**-0.90**
DifferenceAverage	**-0.97**	**-0.97**	**-0.97**	**-0.97**	**-0.97**	**-0.97**	**-0.97**
DifferenceVariance	**-0.86**	**-0.88**	**-0.86**	**-0.87**	**-0.88**	**-0.80**	**-0.86**
DifferenceEntropy	**-0.99**	**-0.99**	**-0.99**	**-0.99**	**-0.99**	**-0.99**	**-0.98**
SumEntropy	**-0.99**	**-0.99**	**-0.99**	**-0.98**	**-0.98**	**-0.97**	**-0.98**
JointEnergy	**0.91**	**0.88**	**0.88**	**0.89**	**0.84**	**0.87**	**0.83**
Contrast	**-0.89**	**-0.90**	**-0.89**	**-0.90**	**-0.90**	**-0.88**	**-0.88**
Id	**0.97**	**0.96**	**0.96**	**0.96**	**0.95**	**0.95**	**0.94**
Idn	0.23	0.19	0.20	0.08	0.20	0.10	0.07
Idm	**0.96**	**0.94**	**0.95**	**0.94**	**0.93**	**0.93**	**0.91**
Idmn	0.19	0.15	0.10	0.05	0.21	0.09	0.04
InverseVariance	**0.87**	**0.84**	**0.82**	**0.80**	**0.84**	**0.76**	**0.79**
Correlation	0.03	-0.12	0.02	-0.10	0.04	-0.19	-0.18
Autocorrelation	**-0.88**	**-0.89**	**-0.88**	**-0.90**	**-0.90**	**-0.86**	**-0.85**
ClusterTendency	**-0.76**	**-0.78**	**-0.74**	**-0.77**	**-0.78**	**-0.78**	**-0.74**
ClusterShade	**-0.30**	**-0.32**	**-0.29**	**-0.31**	**-0.33**	**-0.34**	**-0.30**
ClusterProminence	**-0.30**	**-0.34**	**-0.28**	**-0.31**	**-0.35**	**-0.36**	**-0.29**
Imc1	**0.89**	**0.90**	**0.90**	**0.91**	**0.91**	**0.93**	**0.90**
Imc2	**-0.86**	**-0.86**	**-0.85**	**-0.84**	**-0.82**	**-0.80**	**-0.79**

**NGTDM**	**1.80**	**1.90**	**2.00**	**2.10**	**2.20**	**2.30**	**2.40**
Busyness	**0.82**	**0.78**	**0.77**	**0.82**	**0.78**	**0.70**	**0.75**
Coarseness	0.17	0.28	0.22	0.27	0.20	0.18	0.27
Complexity	**-0.73**	**-0.79**	**-0.76**	**-0.79**	**-0.81**	**-0.78**	**-0.80**
Contrast	**-0.80**	**-0.84**	**-0.77**	**-0.79**	**-0.76**	**-0.78**	**-0.74**
Strength	**-0.67**	**-0.74**	**-0.72**	**-0.76**	**-0.72**	**-0.76**	**-0.76**

**GLRLM**	**1,80**	**1,90**	**2,00**	**2,10**	**2,20**	**2,30**	**2,40**
GrayLevelNonUniformity	**0.95**	**0.94**	**0.95**	**0.94**	**0.94**	**0.93**	**0.92**
GrayLevelNonUniformityNormalized	**0.97**	**0.96**	**0.96**	**0.95**	**0.95**	**0.95**	**0.94**
GrayLevelVariance	**-0.79**	**-0.82**	**-0.79**	**-0.82**	**-0.84**	**-0.82**	**-0.80**
HighGrayLevelRunEmphasis	**-0.89**	**-0.90**	**-0.89**	**-0.91**	**-0.90**	**-0.87**	**-0.87**
LongRunEmphasis	**0.73**	**0.62**	**0.72**	**0.60**	**0.54**	**0.56**	**0.50**
LongRunHighGrayLevelEmphasis	**-0.88**	**-0.89**	**-0.89**	**-0.90**	**-0.90**	**-0.87**	**-0.86**
LongRunLowGrayLevelEmphasis	**0.40**	0.22	0.26	**0.28**	**0.34**	0.24	**0.27**
LowGrayLevelRunEmphasis	**0.42**	0.25	**0.28**	**0.34**	**0.36**	**0.33**	0.27
RunEntropy	**-0.95**	**-0.94**	**-0.95**	**-0.94**	**-0.94**	**-0.93**	**-0.93**
RunLengthNonUniformity	**-0.68**	**-0.64**	**-0.73**	**-0.61**	**-0.56**	**-0.57**	**-0.51**
RunLengthNonUniformityNormalized	**-0.68**	**-0.63**	**-0.73**	**-0.61**	**-0.56**	**-0.54**	**-0.50**
RunPercentage	**-0.72**	**-0.65**	**-0.74**	**-0.62**	**-0.56**	**-0.57**	**-0.51**
RunVariance	**0.70**	**0.53**	**0.66**	**0.53**	**0.47**	**0.51**	**0.45**
ShortRunEmphasis	**-0.68**	**-0.63**	**-0.73**	**-0.61**	**-0.56**	**-0.54**	**-0.51**
ShortRunHighGrayLevelEmphasis	**-0.89**	**-0.90**	**-0.89**	**-0.91**	**-0.90**	**-0.87**	**-0.86**
ShortRunLowGrayLevelEmphasis	**0.42**	0.25	**0.28**	**0.35**	**0.37**	**0.33**	0.26

**GLSZM**	**1.80**	**1.90**	**2.00**	**2.10**	**2.20**	**2.30**	**2.40**
GrayLevelNonUniformity	**0.82**	**0.80**	**0.83**	**0.78**	**0.85**	**0.79**	**0.80**
GrayLevelNonUniformityNormalized	**0.95**	**0.93**	**0.94**	**0.92**	**0.93**	**0.92**	**0.92**
GrayLevelVariance	**-0.77**	**-0.81**	**-0.78**	**-0.80**	**-0.83**	**-0.81**	**-0.82**
HighGrayLevelZoneEmphasis	**-0.88**	**-0.90**	**-0.89**	**-0.91**	**-0.90**	**-0.87**	**-0.87**
LargeAreaEmphasis	**0.67**	**0.59**	**0.69**	**0.54**	**0.51**	**0.51**	**0.44**
LargeAreaHighGrayLevelEmphasis	**-0.68**	**-0.74**	**-0.69**	**-0.73**	**-0.80**	**-0.70**	**-0.68**
LargeAreaLowGrayLevelEmphasis	**0.36**	0.21	0.24	0.23	**0.33**	0.18	**0.29**
LowGrayLevelZoneEmphasis	**0.44**	**0.28**	**0.35**	**0.38**	**0.41**	**0.29**	**0.28**
SizeZoneNonUniformity	**-0.67**	**-0.59**	**-0.74**	**-0.57**	**-0.50**	**-0.54**	**-0.54**
SizeZoneNonUniformityNormalized	**-0.63**	**-0.52**	**-0.68**	**-0.53**	**-0.44**	**-0.48**	**-0.50**
SmallAreaEmphasis	**-0.62**	**-0.52**	**-0.67**	**-0.54**	**-0.44**	**-0.47**	**-0.51**
SmallAreaHighGrayLevelEmphasis	**-0.87**	**-0.89**	**-0.89**	**-0.91**	**-0.89**	**-0.86**	**-0.86**
SmallAreaLowGrayLevelEmphasis	**0.29**	0.16	0.25	**0.31**	**0.35**	0.17	0.17
ZoneEntropy	**-0.66**	**-0.62**	**-0.60**	**-0.62**	**-0.65**	**-0.57**	**-0.62**
ZonePercentage	**-0.72**	**-0.63**	**-0.75**	**-0.63**	**-0.53**	**-0.56**	**-0.53**
ZoneVariance	**0.58**	**0.49**	**0.59**	**0.43**	**0.45**	**0.38**	**0.31**

**GLDM**	**1.80**	**1.90**	**2.00**	**2.10**	**2.20**	**2.30**	**2.40**
DependenceEntropy	**-0.66**	**-0.58**	**-0.53**	**-0.56**	**-0.63**	**-0.52**	**-0.54**
DependenceNonUniformity	**-0.69**	**-0.63**	**-0.75**	**-0.61**	**-0.53**	**-0.57**	**-0.53**
DependenceNonUniformityNormalized	**-0.70**	**-0.63**	**-0.76**	**-0.61**	**-0.53**	**-0.56**	**-0.51**
DependenceVariance	**0.52**	**0.50**	**0.55**	**0.44**	**0.40**	**0.37**	**0.31**
GrayLevelNonUniformity	**0.95**	**0.93**	**0.95**	**0.94**	**0.93**	**0.93**	**0.92**
GrayLevelVariance	**-0.80**	**-0.82**	**-0.79**	**-0.82**	**-0.83**	**-0.82**	**-0.79**
HighGrayLevelEmphasis	**-0.89**	**-0.90**	**-0.89**	**-0.91**	**-0.90**	**-0.87**	**-0.86**
LargeDependenceEmphasis	**0.69**	**0.62**	**0.70**	**0.58**	**0.55**	**0.53**	**0.46**
LargeDependenceHighGrayLevelEmphasis	**-0.70**	**-0.74**	**-0.67**	**-0.69**	**-0.77**	**-0.69**	**-0.66**
LargeDependenceLowGrayLevelEmphasis	**0.33**	0.19	0.20	0.12	0.26	0.15	0.24
LowGrayLevelEmphasis	**0.40**	0.23	0.25	**0.32**	**0.35**	**0.30**	0.26
SmallDependenceEmphasis	**-0.70**	**-0.62**	**-0.75**	**-0.61**	**-0.51**	**-0.54**	**-0.53**
SmallDependenceHighGrayLevelEmphasis	**-0.87**	**-0.88**	**-0.88**	**-0.90**	**-0.89**	**-0.85**	**-0.84**
SmallDependenceLowGrayLevelEmphasis	0.26	0.15	0.19	**0.28**	**0.32**	0.20	0.14
